# Rod-like hybrid nanomaterial with tumor targeting and pH-responsive for cancer chemo/photothermal synergistic therapy

**DOI:** 10.1186/s12951-022-01527-1

**Published:** 2022-07-16

**Authors:** Shaochen Wang, Qiaoqiao Zhou, Shuling Yu, Shuang Zhao, Jiahua Shi, Jintao Yuan

**Affiliations:** 1 Key Laboratory of Natural Medicine and Immune-Engineering of Henan Province, Henan University, Kaifeng, Henan 475004 People’s Republic of China; 2 College of Public Health, Zhengzhou University, Zhengzhou, 450001 People’s Republic of China

**Keywords:** Polyrotaxane, Gold nanorods, Targeted synergistic therapy, pH-Responsive, Hybrid nanomaterials

## Abstract

The development of chemo/photothermal nanotherapeutic systems with excellent photothermal performance, stable drug loading, tumor targeting and strong membrane penetration still remains a challenge. To address this problem, herein a rod-like nanocomposite system (AuNR@FA-PR/PEG) forming from folic acid (FA) terminated carboxylated cyclodextrin (CD) pseudopolyrotaxane (FA-PR) and polyethylene glycol (PEG) modifying gold nanorods (AuNR) was reported. Cisplatin (CDDP) was loaded in AuNR@FA-PR/PEG via coordination bonds to prepare a rod-like pH-responsive nanosystem (AuNR@FA-PR/PEG/CDDP) with chemotherapy/photothermal therapy. The rod-like morphology of AuNR@FA-PR/PEG was characterized by transmission electron microscope. In vitro drug release experiments showed the pH-responsive of AuNR@FA-PR/PEG/CDDP. In vivo real-time imaging assays proved AuNR@FA-PR/PEG/CDDP could rapidly enrich in the tumor area and stay for a long time because of folate targeting and their rod-like morphology. In vivo photothermal imaging assays showed AuNR@FA-PR/PEG/CDDP excellent photothermal performance, the average temperature of tumor region could reach 63.5 °C after 10 min irradiation. In vitro and in vivo experiments also demonstrated that the combined therapy of chemotherapy and photothermal therapy had an outstandingly synergistic effect and improved the therapeutic efficacy comparing with chemotherapy and photothermal therapy alone. Therefore, the prepared rod-like AuNR@FA-PR/PEG/CDDP will provide a new strategy for the effective treatment of cancer.

**Supplementary Information:**

The online version contains supplementary material available at 10.1186/s12951-022-01527-1.

## Introduction

Cancer incidence and mortality increase rapidly worldwide, making cancer the major cause of death in most regions [1]. Chemotherapy plays an important role in cancer treatment. However, the limitations of single chemotherapy, such as drug resistance, incomplete tumor elimination have greatly compromised the therapeutic outcome [2]. In order to improve treatment effects, many other treatment strategies, such as radiotherapy [3], immunotherapy, [4, 5] photothermal therapy (PTT) [6] have been combined with chemotherapy to form dual-modal synergistic therapy for cancer treatment. Specially, PTT can not only directly burn tumor but also assist the efficacy of chemotherapy [7]. The combination treatment of PTT and chemotherapy has been widely researched and become a promising therapeutic therapy [8]. But the effect of PTT-chemotherapy is still restricted to the morphology of carriers and the stability of drug-load in carriers.

AuNR as an excellent PTT agent has shown strongly absorb light in the NIR region and impressive therapeutic effect. [9] Moreover, the surface of AuNR is easily functionalized with other materials such as nucleic acids, amphiphilic polymers and proteins for modifying its surface property [10]. Consequently, many spherical AuNR systems have been formed [11, 12]. However, rod-like nanomaterials can enhance cell membrane penetration, reduce macrophage internalization, prolong circulation time compared with spherical counterparts [13–15]. Therefore, that AuNR is modified and still maintains rod-shaped is crucial for the effective combination therapy of cancer.

PEG and CDs have been widely used in the modification of nanomaterials because both of them can improve the solubility and biocompatibility of nanomaterials, prolong circulation time in the organism [16, 17]. Folic acid (FA) terminated α-CD pseudopolyrotaxane (PR) is a supramolecular macrocyclic polymer consisting of multiple α-CD rings threaded by a linear PEG chain terminated with single end by FA as bulky capping groups. As we all known, FA receptors were distinctively expressed on the surface of tumor cells. FA is used as bulky capping groups not only block the CD on linear axis, but also enhance the tumor targeting of FA-PR. In addition, the nanoscale sizes of PR prepared by us are very small (about 2–4 nm) [18, 19], which is much smaller than that of AuNRs (Length from 20 to 200 nm, width from 5 to 100 nm) [20]. If AuNRs modified by small size FA-PRs would maintain their rod-shaped appearance [21], and enhance their targeting. On the other hand, CDs or modified CDs in the PR have many reactive groups such as hydroxyl, amino or carboxyl group, which can load drug through covalent bonds to make the loaded drug stable in the physiological environment. [18] Specially, modified CDs can move freely on the axle, which can effectively enhance the interactions of PRs and cells by adjusting the CD position to fit external changes. [22–24] Therefore, the hybrid nanomaterials formed from AuNR and FA-PR will simultaneously enhance the stability of drug-loaded in nanomaterials and the interaction with cancer cells.

Cisplatin (CDDP) as an anticancer drug has effects on a variety of cancers, but its non-specific transmission during cycling is more harmful to normal organs and tissues [25]. The microenvironment of tumor is acidic, which is a feature that distinguishes it from normal tissues. The pH-responsive drug-loading nanomaterials are designed to prevent premature release of drugs during circulation in the body and reduce the toxic side effects of drug toward the organism. Therefore, CDDP is loaded in tumor targeting FA-PR/AuNR hybrid nanomaterials through a pH-responsive coordination bond between CDDP and CD in PR to reduce its toxicity to organisms and achieve chemo/photothermal synergistic therapy.

**Scheme 1:**
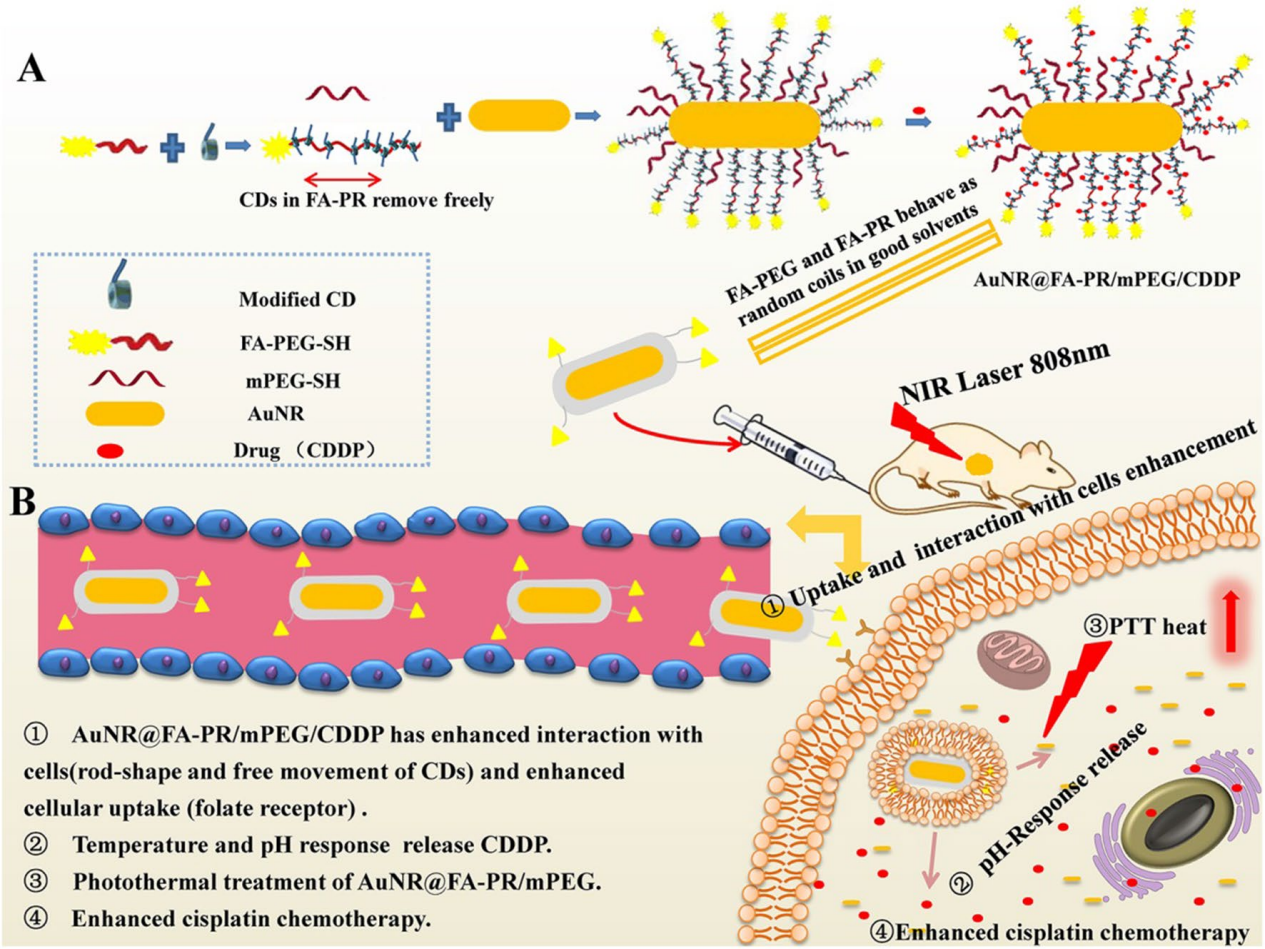
**A** Preparation of AuNR@FA-PR/PEG/CDDP; **B** Mechanism of photothermal therapy (PTT)/chemotherapy (CT) synergistic treatment of cancer with AuNR@FA-PR/PEG/CDDP

The aim of this work is to prepare a pH-responsive, tumor targeting rod-like drug-loaded hybrid delivery system based on FA-PR and AuNRs for achieving the effective targeting and chemotherapy/photothermal synergistic therapy. Firstly, a rod-like hybrid nanomaterial (AuNRs@FA-PR/PEG) was prepared with FA-PRs and PEG modifying AuNRs. CDDP as an antitumor chemotherapy drug is loaded in AuNRs@FA-PR/PEG through pH-responsive coordination bonds to prepare AuNRs@FA-PR/PEG/CDDP. Secondly, the release experiment of drug loaded in AuNRs@FA-PR/PEG/CDDP was tested under physiological environment, acidic conditions, and in vitro experiment. And the photothermal performance of AuNRs@FA-PR/CDDP was studied, too. Finally, the targeting and synergistic anti-tumor effects of chemotherapy and photothermal therapy was studied by in vivo and in vitro experiments. The preparation schematic of AuNR@FA-PR/PEG and the mechanism of photothermal therapy (PTT)/chemotherapy (CT) synergistic treatment of cancer with AuNR@FA-PR/PEG/CDDP were showed in scheme 1.

## Experiment section

### Synthesis of FA-PEG4000-SH

FA (0.75 mol, 331.05 mg) was dissolved in 10 mL of dried DMSO, EDC (0.48 mmol, 92.02 mg) and NHS (0.48 mmol, 55.25 mg) were rapidly added to the system, and then the system reacted for 2 h at 37 °C in the dark. The above solution was slowly added dropwise into a solution of PEG4000 (0.2 mmol, 800 mg) in DMSO. The reaction continued for 24 h at room temperature. Meanwhile, EDC (0.8 mmol, 153.36 mg) and NHS (0.8 mmol, 92.08 mg) were added to a solution containing 3-mercaptopropionic acid (0.4 mmol, 42.46 mg) in 5 mL of DMSO, which was activated at 37 °C with stirring, and then the solution was added to the above solution. The system continued stirring for 48 h at room temperature. Finally, the reaction stopped and the solution was dialyzed with a dialysis membrane (3.5 kDa) against distilled water for 48 h to remove the solvent and unreacted materials. The free FA in dialysis solution was removed by centrifugation at 16,000 rpm to give a pure pale yellow solution. The pure pale yellow solution was lyophilized to give a pale yellow fluffy solid.

### Preparation of water-solution targeting pseudopolyrotaxane (FA-PR-SH)

The pseudopolyrotaxane was formed by self-assembly of carboxylated cyclodextrin (Detailed preparation process was presented in support information) and FA-PEG-SH. The prepared α-CD-COOH (60.8 mg, 0.044 mmol) was dissolved in as little deionized water as possible, then FA-PEG-SH (20 mg, 0.0044 mmol) was added to the above solution, and then the system was sonicated for 10 min. Finally, the reaction continued for 36 h at room temperature in the dark to obtain FA-PR. The reaction solution was processed by dialysis, and the dialysate was lyophilized to collect the product.

### Preparation of FA-PR modified AuNR hybrid nanomaterials (AuNR@FA-PR/PEG)

The AuNRs were prepared with the method reported in literature [20] and the detail preparation process was presented in supporting information. The formation of hybrid nanomaterials (AuNR@FA-PR/PEG) was through the Au-S bond of and FA-PR-SH, mPEG-SH and AuNR. Specifically, a solution of AuNR (1 mL, 1 mg mL^−1^) was added to FA-PR-SH (5 mL, 5 mg mL^−1^), the reaction system continued reacting with stirring for 24 h at room temperature. After that the reaction was stopped and the solution was centrifuged to get AuNR@FA-PR. The sulfhydrylation of mPEG is mainly carried out by the method reported in the literature [26]. The detailed preparation process was presented in supporting information. The prepared AuNR@FA-PR was dispersed in mPEG-SH (2 mL, 0.75 mg mL^−1^) solution, and the mixture reacted for 36 h at room temperature, finally the reaction was stopped and the solution was centrifuged to collect precipitation, the precipitation was washed twice with deionized water to obtain AuNR@FA-PR/PEG. AuNR@FA-PR/PEG was suspended in deionized water and stored in a refrigerator at 4 °C.

### The morphology and structures of AuNRs and AuNR@FA-PR/PEG

The morphology of AuNR and AuNR@FA-PR/PEG was mainly characterized by transmission electron microscopy (TEM). In detail, the sample was dissolved in distilled water, and dispersed uniformly by ultrasound, and 6 µL of solution is taken out with a pipette. The solution was dropped on ordinary carbon films, and naturally dried at room temperature, finally, the morphology was observed by a transmission electron microscopy.

In order to further prove the successful modification of AuNR, the Fourier transform infrared spectroscopy (FT-IR) of AuNR and AuNR@FA-PR/PEG were detected. In detail, small amount of sample was mixed with dry potassium bromide, and fully ground with an agate mortar. Then the mixture was put in a clean compression mold (spread evenly in the compression mold), and pressed using a tablet press for 1–2 min under a pressure of 20 MPa into a transparent sheet, which can be used for measurement. The prepared materials were also measured by an ultraviolet spectrophotometer (UV-vis). The samples were dissolved in distilled water to prepare the same concentration of an aqueous solution. The distilled water was used as a blank control, and the absorbance at 200–1000 nm was measured.

### The preparation of drug-loaded AuNR@FA-PR/PEG (AuNR@FA-PR/PEG/CDDP)

The surface of the AuNR@FA-PR/PEG is rich in carboxyl groups, thus CDDP can be loaded in the hybrid nanomaterials through chelation bonds. The detailed procedure is as follows: 25 mg of AuNR@FA-PR/PEG was resuspended in 5 mL of deionized water, and then 3 mg of CDDP was added into the system, the samples were uniformly mixed by ultrasound. The system was placed on a shaker for 48 h in the dark. After that, the reaction solution was centrifuged, and the precipitation was collected and washed twice with distilled water to remove unreacted CDDP. The product AuNR@FA-PR/PEG/CDDP was obtained and stored at 4 °C for use. Unreacted CDDP was measured by the o-phenylenediamine colorimetric method mentioned in reported reference [19], and the drug loading content was calculated to be about 7.4% by using Eq. (1).

1 $${\text{DLC }}(\% ) \, = \, \frac{{\text{Weight of drug in hybrid nanomaterials }}}{{\text{Weight of hybrid nanomaterials }}} \, \times \, 100\% \,$$

### The release of CDDP loaded in AuNR@FA-PR/PEG/CDDP

AuNR@FA-PR/PEG/CDDP (74 μg/mL CDDP, 1 mL) was placed in a 3.5 KDa dialysis bag and immersed in 20 mL of PBS solution with various pH values (7.4, 6.5, 5) in 50 mL centrifuge tubes. The centrifuge tubes were placed in a shaker with room temperature at a speed of 150 r/min, and 1 mL of dialysate was taken at predetermined times (0.25, 1, 2, 4, 8, 12, 24, 48, 72, 96, 120, 168 h), and then 1 mL of new PBS solution with different pH value was added into the corresponding centrifuge tubes. Simultaneously, the photothermal effect dependent drug release of AuNR@FA-PR/PEG/CDDP was carried out. The sample was irradiated with an 808 nm laser (1.5 W cm^−2^) for 10 min in every time stage of drug release. Finally, the absorption value at 703 nm was measured by an ultraviolet spectrophotometer and the drug release was calculated by an accumulation method. The formula is as follows:

2 $$E_{r} = \frac{{V_{e} \sum\limits_{1}^{n - 1} {C_{i} } + V_{0} C_{n} }}{{m_{\text{drug}} }} \times 100\%$$

(Where, E_r_: Cumulative drug release (%); V_e_: Sample volume (mL); V_0_: Total volume of the released medium (mL); C_i_: Concentration of the drug in the i th sample (μg/mL); C_n_: Concentration of the drug in the n th sample (μg/mL); m: Total amount of drug in the drug-loaded micelle (μg)).

### Photothermal performance of AuNR@FA-PR/PEG/CDDP

In order to measure the photothermal conversion capability AuNR@FA-PR/PEG/CDDP, an in vitro photothermal conversion experiment was carried out, which was briefly described as follows: AuNR@FA-PR/PEG/CDDP was prepared into 50 µg mL^−1^ aqueous solution. 1 mL of the solution was taken and placed in a quartz cuvette. Then the solution was irradiated with an 808 nm laser (1.5 W cm^−2^) for 10 min, the change in temperature was recorded every 15 s during the irradiation, and the distilled water is used as a blank control group.

To study the relation between the concentration of AuNR@FA-PR/PEG/CDDP and the rate of heat generation, AuNR@FA-PR/PEG/CDDP solution with various concentration (0, 25, 50, 100, 200 µg mL^−1^) were irradiated with an 808 nm laser (1 W cm^−2^) for 10 min, and the change in temperature was recorded every 10 s during irradiating. Simultaneously, AuNR@FA-PR/PEG/CDDP (50 µg mL^−1^) was exposed to an 808 nm laser with various power densities (0.75, 1.5, 2.25, 3 W cm^−2^), and the temperature change at the scheduled irradiation time point is recorded.

In order to further investigate the photothermal stability of AuNR@FA-PR/PEG/CDDP, the solution of AuNR@FA-PR/PEG/CDDP (100 µg mL^−1^) was irradiated with an 800 nm laser (1 W cm^−2^) for four cycles of laser on/off irradiation experiment.

s, and every cycle contained a 10 min NIR-light exposure period followed by a period of cooling to room temperature. All of the temperature changes were recorded with a thermocouple device. The photothermal conversion efficiency (η) was calculated as follows:

3 $$\eta = \frac{{hS(T_{\max ,M} - T_{S} )}}{{I(1 - 10^{ - A808} )}}$$

4 $$t = - \tau_{S} {\kern 1pt} {\text{ln(}}\theta {)}$$

5 $$\tau_{S} = \frac{mC}{{hS}}$$

6 $$\theta = \frac{{T - T_{S} }}{{T_{{\max ,{\text{M}}}} - T_{S} }}$$

where m and C are the mass and heat capacity of water, respectively. h and S represent the heat transfer coefficient and the surface area of the container, respectively. I represents the power density of an 808 nm laser. *A*808 is the absorbance of AuNR@FA-PR/PEG/CDDP at 808 nm. T_max,M_ and TS are the maximum temperature during irradiation and room temperature, respectively. τ_s_ is the time constant. The photothermal conversion efficiency was calculated as η = 26.35%.

### Cell uptake experiment

Firstly, AuNR@FA-PR/PEG/CDDP was labeled with fluorescein isothiocyanate (FICT), and the preparation process was as follows: 1 mL solution of FITC dissolved anhydrous DMSO (1.0 mg mL^−1^) was added into 10 mL solution of AuNR@FA-PR/PEG/CDDP, and the system was stirred for 24 h at room temperature under dark condition. After that, the reaction was stopped and the mixture was centrifuged to collect the precipitation, then the precipitation was washed three times with distilled water to remove the unreacted FITC, finally, the FITC-labeled AuNR@FA-PR/PEG/CDDP was redispersed in distilled water for cellular uptake use.

HepG2 cells maintained in DMEM supplemented with 10% fetal bovine serum (FBS), streptomycin (100 U/mL), penicillin (100 U/mL) culture medium were inoculated into a 6-well plate, and placed in a 5% CO_2_ incubator at 37 °C for 24 h. Then, the medium was discarded, and 2 mL of medium solution containing 240 μg of FITC-labeled AuNR@FA-PR/PEG/CDDP was added into the plates and further incubated for 4 h. The medium was aspirated and the cells were washed three times with PBS to remove the free FITC-labeled AuNR@FA-PR/PEG/CDDP. 4% paraformaldehyde was added into the plates and fixed for 10 min, and then the cells were washed three times with PBS and stained with nuclear dye DAPI for 15 min in the dark. Finally, the cells were observed with a confocal laser scanning microscopy (CLSM).

### Cytotoxicity and photothermal cytotoxicity of AuNR@FA-PR/PEG

The cytotoxicity and photothermal cytotoxicity of AuNR and AuNR@FA-PR/PEG were investigated by 1-(4,5-dimethylthiazol-2-yl)-3,5-diphenyl-formazan (MTT) assay. Human liver cancer cells (HepG2) were selected as a cell experimental model. DMEM containing 10% fetal bovine serum was used as a medium, and HepG2 cells were placed in a 96-well plate at 7000 cells per well, 90 µL medium was added into every well and cultured for 24 h, and then 10 µL various concentrations (10, 30, 60, 90, 120 µg mL^−1^) of AuNR, AuNR+Laser, AuNR@FA-PR/PEG and AuNR@FA-PR/PEG+Laser were added into plates, each concentration was designed four replicate wells. The cells in AuNR and AuNR@FA-PR/PEG treatment groups continued culturing for 48 h. After that, MTT in PBS solution (50 µL, 1 mg mL^−1^) (tetramethylazozolium salt) was added into per well, and the cells continued incubating for another 4 h. Then the culture medium was discarded, and DMSO (100 µL/well) was added to each well at 37 °C for 10 min, and the plate was gently shaken on a shaker for 5 min to completely dissolve the crystalline material. The absorbance at 570 nm was measured with a microplate reader.

The cells in AuNR + Laser and AuNR@FA-PR/PEG + Laser treatment groups incubated 24 h, then irradiated with an 808 nm laser (1 W cm^−2^) for 3 min, and continued to cultivate for 24 h. MTT (50 µL, 1 mg mL^−1^) (tetramethylazozolium salt) in PBS solution was added into each well. The culture medium was discarded, and 100 µL of DMSO was added to each well to dissolve the crystalline material at 37° C for 10 min. The plate was gently shaken on a shaker for 5 min to completely dissolve the crystalline material, and the absorbance at 570 nm was measured with a microplate reader.

The cell survival rate is calculated as follows:

7 $${\text{Cell viability\% = }}\frac{{{\text{OD}}_{{{\text{sample}}}} \, - {\text{OD}}_{{{\text{blank}}}} }}{{{\text{OD}}_{{{\text{control}}}} - {\text{OD}}_{{{\text{blank}}}} }} \, \times {\text{ 100\% }}$$

### Cytotoxicity and photothermal cytotoxicity of AuNR@FA-PR/PEG/CDDP

HepG2 cells in logarithmic growth phase were seeded at a density of 7000/well in 96-well plates with 90 μL culture medium per well. After incubating for 24 h, various concentrations (1, 2, 5, 8, 10 μg/mL, CDDP) of CDDP, CDDP+Laser, AuNR@FA-PR/PEG/CDDP and AuNR@FA-PR/PEG/CDDP+Laser were added into the plate, and each concentration was designed four replicate wells. The cells in CDDP and AuNR@FA-PR/PEG/CDDP treatment rows continued cultivating for 48 h. After that, 50 µL of 1 mg mL^−1^ MTT in PBS solution was added into the plate, and the cells continued incubating for 4 h, then the medium was removed. 100 µL of DMSO was added into per well, and the plate was maintained at 37 °C for 10 min, then gently shaken to make intracellular crystalline formamidine sufficiently dissolve. Finally, the absorption value was measured at a wavelength of 570 nm using a microplate reader.

The cells in CDDP+Laser and AuNR@FA-PR/PEG/CDDP+Laser treatment rows continued to culture 24 h, and irradiated with an 808 nm laser (1 W cm^−2^) for 3 min per well, then cultivated for another 24 h. 50 µL of MTT solution was added into per well, and the cells continued to culture for 4 h. The medium was removed, and 100 µL of DMSO was added into per well. The pate was maintained at 37 °C for 10 min, and the crystal formamidine was sufficiently dissolved by gently shaking. The absorption was measured at a wavelength of 570 nm using a microplate reader.

To investigate the cytotoxicity of photothermal and chemotherapy to normal cells, human normal liver cells HL-7702 were selected as cell experimental model. DMEM containing 10% fetal bovine serum was used as a medium, and HL-7702 cells were placed in a 96-well plate at 7000 cells per well, 90 µL medium was added into every well and cultured for 24 h, and then 10 µL various concentrations (1, 2, 5, 8, 10 µg mL^−1^) of CDDP, CDDP+Laser, AuNR@FA-PR/PEG/CDDP, AuNR@FA-PR/PEG/CDDP+Laser were added into plates, each concentration was designed four replicate wells. The cells in CDDP and AuNR@FA-PR/PEG/CDDP treatment groups continued culturing for 48 h. The cells in CDDP + Laser and AuNR@FA-PR/PEG/CDDP+Laser treatment groups were incubated 24 h, then irradiated with an 808 nm laser (1 W cm^−2^) for 3 min, and continued to cultivate for another 24 h. After that, MTT in PBS solution (50 µL, 1 mg mL^−1^) (tetramethylazozolium salt) was added into per well, and the cells continued incubating for another 4 h. Then the culture medium was discarded, and DMSO (100 µL/well) was added to each well, and the plate was gently shaken on a shaker for 5 min to completely dissolve the crystalline material. The absorbance at 570 nm was measured with a microplate reader. The survival rate was calculated, and the average value was measured three times. The survival rate was also calculated by Eq. (7).

### Labeling AuNR@FA-PR/PEG/CDDP with NIR-797 isothiocyanate and in vivo real-time imaging

Firstly, AuNR@FA-PR/PEG/CDDP was labeled with NIR-797 Isothiocyanate (NIR797), and the preparation process was as follows: 1 mL solution of NIR-797 isothiocyanate dissolved anhydrous DMSO (1.0 mg mL^−1^) was added into 10 mL solution of AuNR@FA-PR/PEG/CDDP, and the system was stirred for 24 h at room temperature under dark conditions. After that, the reaction was stopped and the mixture was centrifuged to collect the precipitation, then the precipitation was washed three times with distilled water to remove the unreacted NIR797, finally, the NIR797-labeled AuNR@FA-PR/PEG/CDDP was redispersed in distilled water for real-time imaging.

All the protocols for the animal tests have been reviewed and approved by the Animal Management and Ethics Committee of Henan University (Nos: HUSOM-2019-010) and performed in accordance with the guidelines provided by the National Institute of Animal Care. The murine H22 tumor-bearing Kunming mice models were established by inoculating subcutaneously 5 × 10^5^ cells into the right armpit region of female mice. The mouse was kept with free access to food and water. When the tumor volume reached about 120 mm^3^, 0.2 mL of NIR797-labeled AuNR@FA-PR/PEG/CDDP were injected into tumor-bearing mice through a tail vein. The real-time distribution of NIR797-labeled AuNR@FA-PR/PEG/CDDP in tumor-bearing mice was imaged using an IVIS Lumina XRMS Series (USA, PerkinElmer) in vivo imaging system. The mice were anesthetized at 168 h after the tail vein injection. Finally, the main organics (heart, liver, spleen, lung, kidney, stomach, intestines and brain) and tumor were collected for isolated organ imaging to investigate the accumulation of AuNR@FA-PR/PEG/CDDP in different organs and tumors.

### The accumulation of AuNR@FA-PR/PEG/CDDP in Tumor at different times

Real-time Imaging of NIR-797-labled AuNR@FA-PR/PEG/CDDP in tumor was carried out. Eighteen H22 tumor-bearing mice were randomly divided into six groups with 3 mice in each group. 0.2 mL NIR-797-labled AuNR@FA-PR/PEG/CDDP was intravenously injected into each mouse at a dose of 5 mg/kg (CDDP). Then six groups of mice were sacrificed at predetermined time points (6 h, 12 h, 24 h, 48 h, 72 h, 96 h), respectively, the tumors were harvested and weighed. Finally, the tumors were imaged using an IVIS Lumina XRMS Series.

Simultaneously, the collected tumors were digested in a mixed acid solution (perchloric acid: concentrated nitric acid, 1:3). Then the solution was heated at 100 ℃ for 24 h, the white crystal appears in the bottom and was dissolved with 5% dilute hydrochloric acid, and then the solution was made up to 2 ml. The ICP-AEs test was used to determine the Au element content in each tumor. Finally, the accumulation of AuNR@FA-PR/PEG/CDDP in tumor at different time points was calculated. The data were normalized to the tissue weight and expressed as percentage of dose/g at each test point.

### Hemolysis assay

The eyeball blood was obtained from healthy Kunming mice, and then centrifuged to collect red blood cells. In detail, red blood cells were separated by centrifugation at 2000 rpm for 3 min, washed with 0.9% NaCl solution until the supernatant was colorless, and then separately mixed with 1 mL of water (negative control), saline (positive control), and AuNR@FA-PR/PEG/CDDP solution (12.5, 25, 50, 100, 200 µg mL^−1^). After incubating at 37 °C for 3 h, the supernatants were collected by centrifugation and the absorbance at 540 nm was recorded using a UV-vis spectrophotometer. The percentage of hemolysis was calculated by the following equation:

8 $${\text{Hemolysis (\%) = }}\frac{{{\text{Abs}}_{{{\text{sample}}}} \, - {\text{Abs}}_{{{\text{negative control}}}} }}{{{\text{Abs}}_{{{\text{positive control}}}} - {\text{Abs}}_{{{\text{negative control}}}} }} \, \times {\text{ 100\% }}$$

where Abs_sample_, Abs_negative_, and Abs_positive_ are the absorbance of samples, the negative control, and the positive control, respectively.

### In vitro photothermal imaging

AuNR@FA-PR/PEG/CDDP was dissolved in distill water to prepare a solution with 50 µg mL^−1^ of concentration, and the solution was subjected to laser irradiation with different laser powers (0.75, 1.5, 2.25, 3 W cm^−2^) for 10 min. At the same time, AuNR@FA-PR/PEG/CDDP was also prepared various concentration water solution and subjected to an 808 nm laser irradiation with 1 W cm^−2^ of power for 10 min. The temperature change of AuNR@FA-PR/PEG/CDDP solution was recorded with an infrared thermal imaging camera (Thermo Shot F30, Nippon Avionics Co., Ltd, Japan), and the temperatures at tumor sites were measured.

### In vivo temperature measurement and photothermal imaging

According to the in vivo fluorescence imaging of mice injected with NIR-797-labled AuNR@FA-PR/PEG/CDDP, the nanocomposite began to enter and accumulate in the tumor area at 6 h after the injection of AuNR@FA-PR/PEG/CDDP. Therefore, the thermal imaging of the tumor regions in H22 tumor-bearing mice was carried out at 6 h after injection of various samples through the tail vein to verify the accumulation and photothermal capabilities of AuNR@FA-PR/PEG/CDDP. The H22 tumor-bearing mice were divided into three groups when the tumor sizes reached about 120 mm^3^. Three groups mice were treated with saline, AuNR@FA-PR/PEG (Au: 55 mg of Au/kg) and AuNR@FA-PR/PEG/CDDP solution (Au: 55 mg of Au/kg), through the tail vein, respectively. At 24 h after injection, the tumors were irradiated with an 808 nm laser (1.0 W cm^−2^) for 10 min. The thermal imaging was captured every 2 min by using an infrared thermal imaging camera (Thermo Shot F30, Nippon Avionics Co., Ltd, Japan), and the temperatures at tumor sites were measured.

### In vivo antitumor efficacy

The tumor models were established by implanting 5 × 10^5^ murine hepatoma cell line H22 into the right armpit of Kunming mice (6−8 weeks). The antitumor activity of AuNR@FA-PR/PEG/CDDP was investigated using the established models. The “day 1” was determined when the tumor volume reached an average size of 90–100 mm^3^. On day 1, tumor-bearing mice were randomly divided into six groups. Saline and AuNR@FA-PR/PEG treatment groups were used as control groups. Two groups of mice were treated with saline and free CDDP (3 mg/kg), two groups of mice were injected with AuNR@FA-PR/PEG (50 mg/kg, 7.5 mg AuNR eq.) through the tail vein, the last two groups of mice were injected with AuNR@FA-PR/PEG/CDDP (50 mg/kg, 7.5 mg AuNR eq., 1.5 mg/kg cisplatin eq.) via the tail vein. The tail vein treatment was performed on the day 1, day 4 and day 7. At 24 h after tail vein injection, One of AuNR@FA-PR/PEG treatment group and one of AuNR@FA-PR/PEG/CDDP treatment group were irradiated using an 808 nm laser (1.0 W cm^−2^) for 5 min. Tumor sizes were measured in two dimensions every other day using a vernier caliper up to 14 days. Simultaneously, the weights of tumor-bearing mice were monitored. The tumor volume was calculated according to the following formula: V = d^2^ × D/2 (where d was the tumor width at the shortest dimension, and D was the longest dimension). On the 15th day, tumor-bearing mice in all treatment groups were sacrificed and the tumors were harvested. The antitumor activity and biocompatibility were evaluated via tumor growth and terminal tumor weight. The tumor growth inhibition (TGI) was calculated by the following formula:

9 $${\text{TGI = }}\frac{{\overline{{\text{V}}} {\text{ of saline control group - }}\overline{{\text{V}}} {\text{ of tested group}}}}{{\overline{{\text{V}}} {\text{ of saline control group}}}} \, \times {\text{ 100\% }}$$

### Determination of cell apoptosis and proliferation and histology studies

Six group tumor-bearing mice were injected saline, CDDP, AuNR@FA-PR/PEG (two groups) and AuNR@FA-PR/PEG/CDDP (two groups) via tail vein, respectively. One AuNR@FA-PR/PEG treatment group and one AuNR@FA-PR/PEG/CDDP treatment group were irradiated with an 808 nm laser for 5 min at 24 h post tail vein injection. After 7 days, all tumor-bearing mice were sacrificed, and the main organs and tumors were harvested, fixed with paraformaldehyde, embedded with paraffin and cut into 4 μm thick sections for hematoxylin and eosin (H&E) staining and examined by optical microscopy.

The apoptotic cells in tumor tissue sections were detected using the Terminal deoxynucleotidyl transferase (TdT)-mediated d-UTP Nick End Labeling (TUNEL) assay (Apoptosis TUNEL assay IHC kit, AbD Serotec, MorphoSys, Oxford, UK, cat.no. APO002) according to the manufacturer’s protocol. The detection of proliferating cells was performed using an antibody against PCNA and the proliferating cells were visualized by incubation with 3,3'-diaminobenzidine tetrahydrochloride (Adamas-beta) for 2 min. After being rinsed with distilled water, the sections were counter-stained with hematoxylin. For quantification of PCNA and TUNEL expression, the number of positive cells was counted in 3 random high power fields (400 × magnification) and divided by the total number of cells for each tumor.

## Statistical analysis

All data were representative results from at least three independent experiments and means ± SEM. The correlation and comparison analyses were performed using the Student’s t-test. *P* values less than 0.05 were considered statistically significant.

## Results and discussion

### Preparation and characterization of AuNR@FA-PR/PEG

**Fig. 1:**
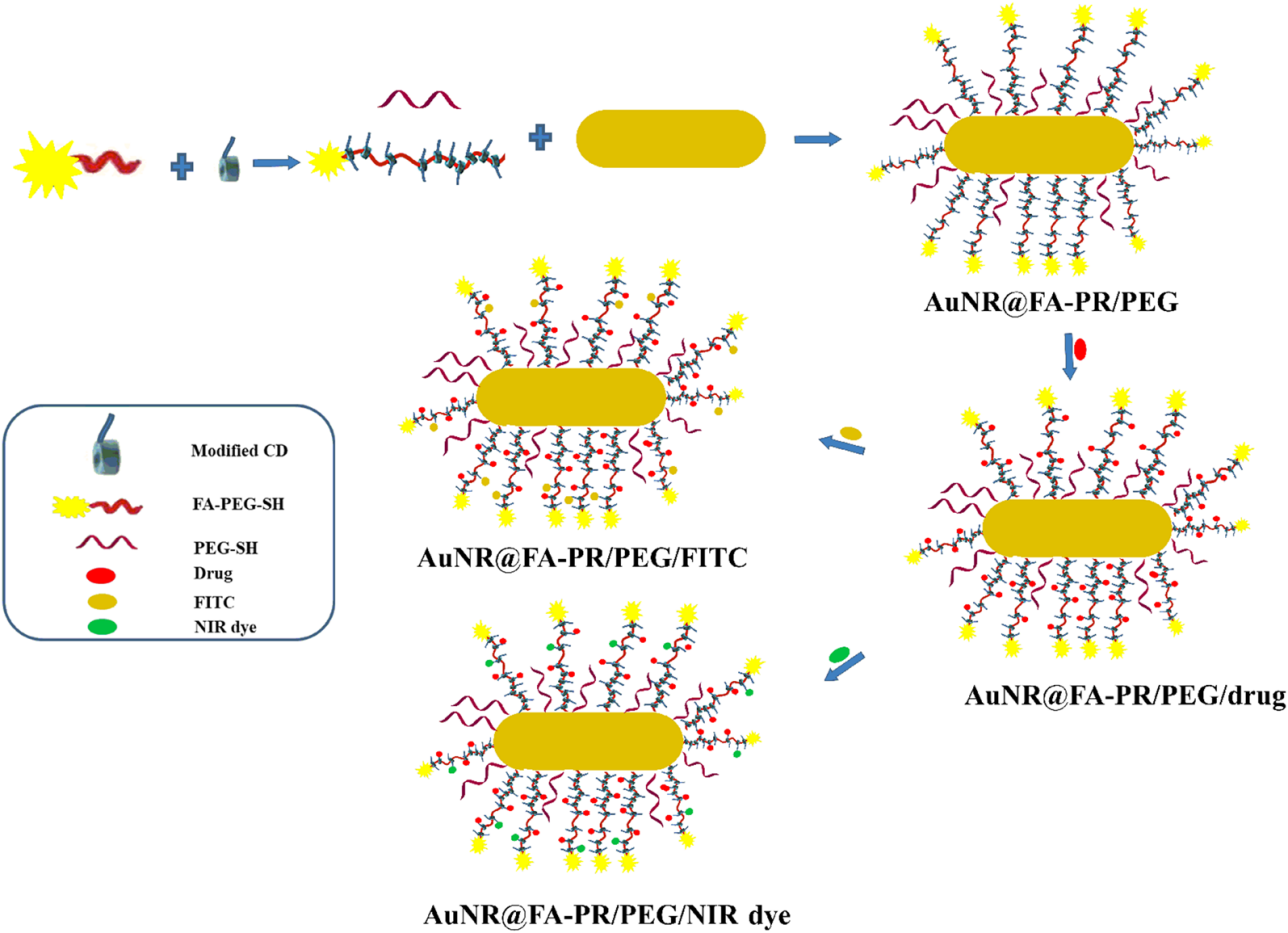
Preparation schematic diagram of hybrid nanosystems

**Fig. 2:**
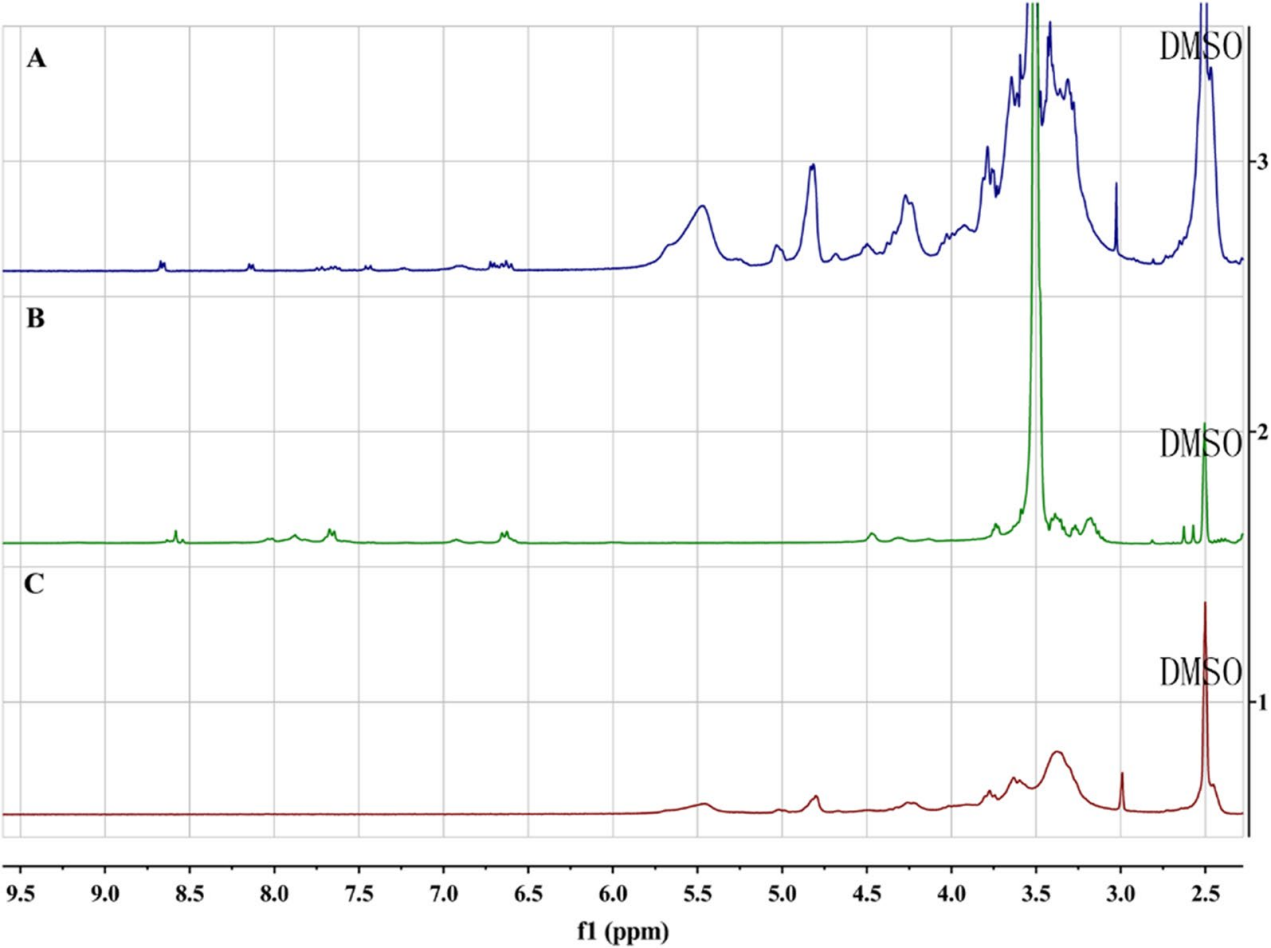
^1^H NMR spectra of FA-PR **A**, FA-PEG-SH **B** and α-CD-COOH **C**

The formation of pseudopolyrotaxane is roughly divided into three parts and the preparation schematic diagram was shown in Fig. 1: (1) the synthesis of FA-PEG4000-SH; (2) the carboxylation modification of α-CD (3) the self-assembly of α-CD-COOH and FA-PEG4000-SH in a homogeneous system by host–guest interaction to form FA-PR-SH. The synthesis compounds were characterized by ^1^H NMR spectra. ^1^H NMR (300 MHz, DMSO-d_6_) spectrum (Fig. 2B) of FA-PEG-SH showed characteristic absorption peaks of FA between δ6.5 and δ8.75, the peak in about δ3.5 was the characterization peak of PEG, which proved the successful preparation of FA-PEG-SH. α-CD-COOH was confirmed successful modification by comparing the ^1^H NMR spectra of α-CD and α-CD-COOH (Additional file 1: Fig. S1). Finally, the ^1^H NMR spectra of FA-PEG-SH, α-CD-COOH and FA-PR-SH were compared and the result was shown in Fig. 2. It can be seen that the FA-PR-SH was successfully prepared by analyzing the spectra of three compounds. In order to further prove the successful preparation of FA-PR-SH, the FT-IR of FA-PEG-SH, α-CD-COOH and FA-PR-SH were provided in the Additional file 1: Fig. S2, 1350 nm and 1154 nm in the spectral of the FA-PR-SH are the characteristic peaks of C-N in FA-PEG-SH and C-O in α-CD-COOH, which could further demonstrate the successful preparation of FA-PR-SH.

### Preparation and characterization of AuNR@FA-PR/PEG and AuNR@FA-PR/PEG/CDDP

**Fig. 3:**
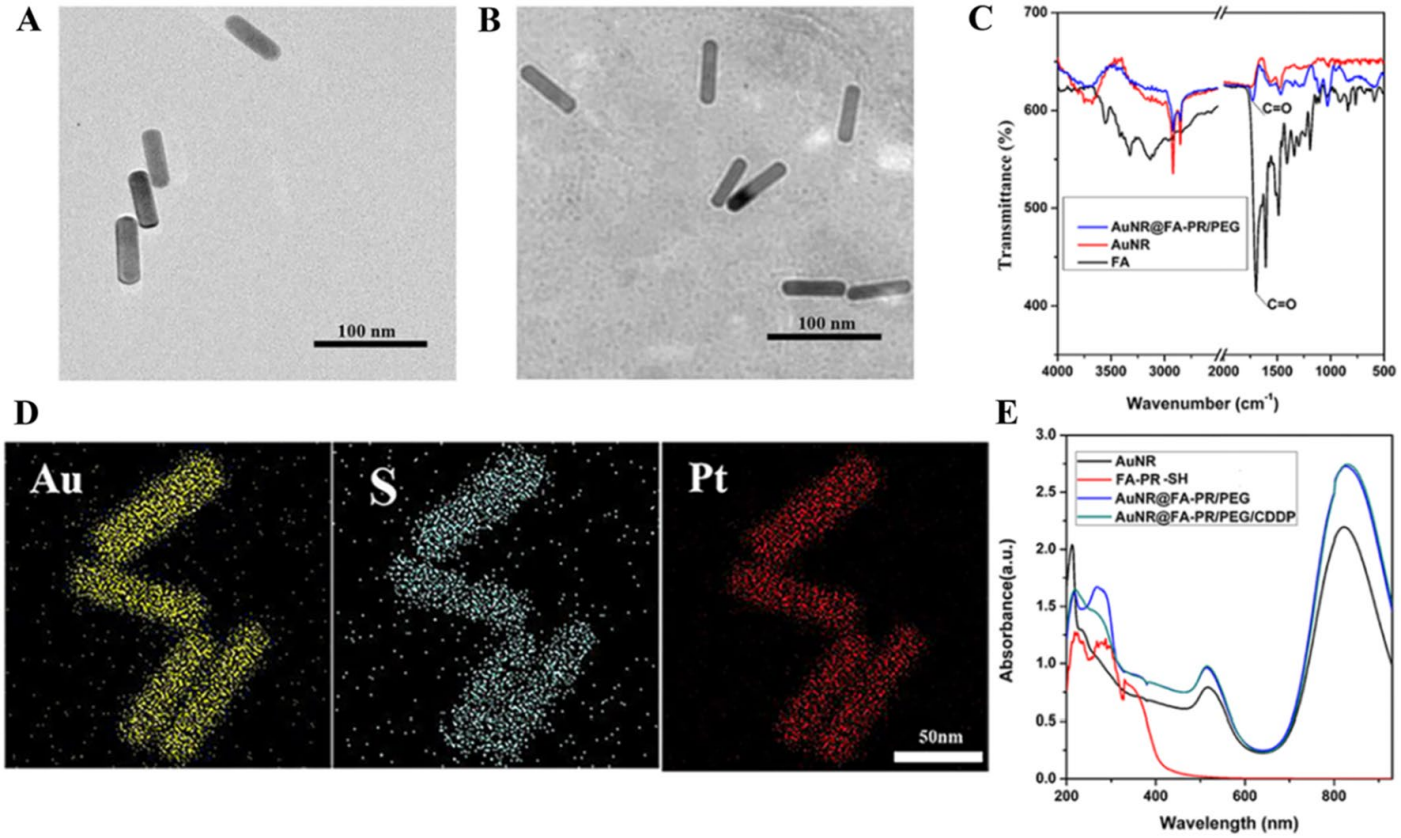
**A** The TEM image of AuNRs; **B** The TEM characterization of AuNR@FA-PR/PEG; **C** FT-IR characterization of FA, AuNR and AuNR@FA-PR/PEG; **D** Elemental mapping images for Au, S and Pt in AuNR@FA-PR/PEG/CDDP ( Scale bar: 50 μm); **E** UV-vis spectral characterization of AuNR, FA-PR-SH, AuNR@FA-PR/PEG and AuNR@FA-PR/PEG/CDDP

The prepared AuNRs were modified with targeting pseudopolyrotaxane and PEG to form stabile, water-soluble and biocompatible composite nanomaterials (AuNR@FA-PR/PEG). The antitumor drug CDDP was loaded in AuNR@FA-PR/PEG by coordination bond to prepare AuNR@FA-PR/PEG/CDDP. The structures and morphologies of AuNRs and AuNR@FA-PR/PEG were characterized with FT-IR, UV–vis spectral and transmission electron microscope (TEM), the corresponding element mapping images of AuNR@FA-PR/PEG/CDDP were provided and the results were shown in Fig. 3. It can be seen from the figure that AuNRs had uniform morphology, uniform particle size and an aspect ratio of about 3.5 (Fig. 3A). In Fig. 3B, it can be found that the surface of AuNRs had obvious modification layer, indicating that AuNR@FA-PR/PEG was successfully prepared, and the prepared hybrid nanomaterials still maintain rod-like morphology. The characteristic elemental mapping of AuNR@FA-PR/PEG/CDDP (Fig. 3D) showed a well-proportioned distribution of Au, S and Pt, which also proved the successful preparation of AuNR@FA-PR/PEG/CDDP. In Fig. 3C, the characteristic peak of FA was observed in FT-IR spectra of AuNR@FA-PR/PEG, the vibration peaks of benzene ring at 3000–3100 nm, 1450 -1650 nm and 900–650 nm, and a stretching vibration peak of carbonyl at about 1700 nm, indicating that FA-PR is successfully combined with AuNRs. Subsequently, the modified AuNR had characteristic absorption peaks of FA at 260 nm, 280 nm and 365 nm, which indicated that AuNR was successfully modified with FA-PR (Fig. 3E).

### In vitro photothermal performance analysis of drug-loaded nanocomposites (AuNR@FA-PR/PEG/CDDP)

**Fig. 4:**
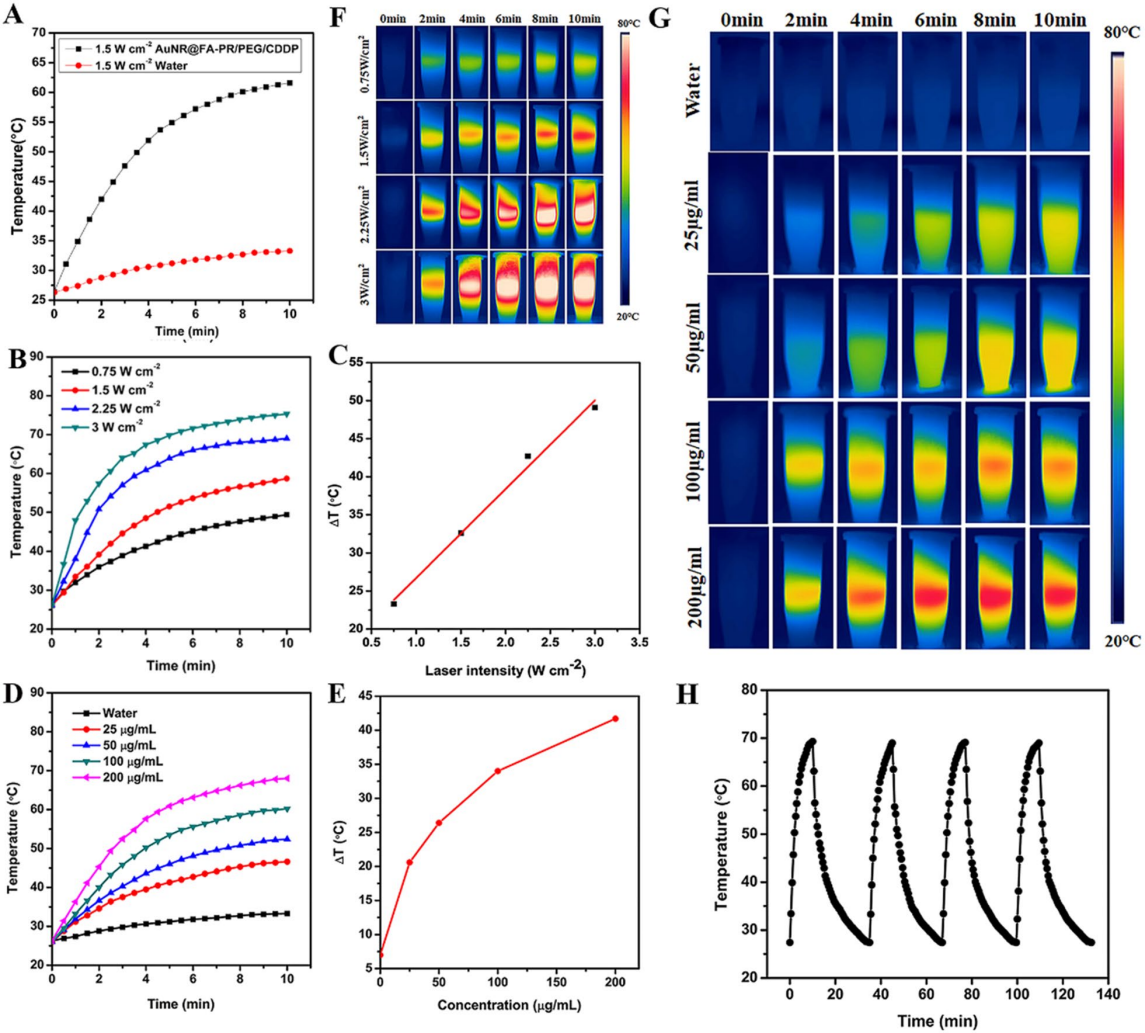
**A** The photothermal heating curves of water and AuNR@FA-PR/PEG/CDDP in (1.5 W cm^−2^, 50 µg mL^−1^); **B** Power density dependent heating curves of AuNR@FA-PR/PEG/CDDP; **C** Maximum temperature elevations (ΔT) of AuNR@FA-PR/PEG/CDDP with different power intensities (50 µg /mL, 0.75, 1.5, 2.25, 3 W cm^−2^); **D** Concentration dependent heating curves of AuNR@FA-PR/PEG/CDDP; **E** Maximum temperature elevations (ΔT) of AuNR@FA-PR/PEG/CDDP with different concentrations (1 W cm^−2^, 0, 25, 50, 100, 200 μg/mL); **F**, **G** Thermal camera images of AuNR@FA-PR/PEG/CDDP with various power intensities (50 µg mL^−1^, 0.75, 1.5, 2.25, 3 W cm^−2^) and concentrations (1 W cm^−2^, 0, 25, 50, 100, 200 µg mL^−1^); **H** Temperature profiles of AuNR@FA-PR/PEG/CDDP (100 μg/mL) during laser on/off operations (808 nm, 1.0 W cm.^−2^)

To investigate the photothermal performance of AuNR@FA-PR/PEG/CDDP, a serial photothermal experiments was carried out, AuNR@FA-PR/PEG/CDDP with predetermined concentration were irradiated with an 808 nm laser (1.5 W cm^−2^) for 10 min, and the distilled water was used as a control group. Figure 4A showed that the temperature of the distilled water group reached 33 ºC, and the temperature change was slight for 7 ºC, while the temperature in AuNR@FA-PR/PEG/CDDP treatment groups could reached 63  C, and the temperature change of AuNR@FA-PR/PEG/CDDP treatment groups was 35 ºC, and the final temperature was up to 63 ºC, which is enough high to kill the cancer cells [27]. The results confirmed that AuNR@FA-PR/PEG/CDDP had an excellent photothermal performance.

For suitable irradiation conditions, the concentration/irradiation intensity-dependent temperature changes were also observed and the results were showed in Fig. 4B–E. In Fig. 4B, C, the temperature change of AuNR@FA-PR/PEG/CDDP solution increases with the enhancement of the laser power and the extension of the irradiation time, the temperature changes were 23, 33, 44, 50 ºC, respectively, corresponding various irradiation power (0.75, 1.5, 2.25, 3 W cm^−2^), and the final temperature reached up to 47, 60, 68, 70 ºC, respectively. The temperature 47 ºC is not enough to kill cancer cells, therefore the tumor cells could not be killed effectively when a laser with 0.75 W cm^−2^ was used to irradiate. The temperatures 68 ºC and 70 ºC may be too high for the normal tissues, so irradiating by lasers with 2.25 and 3 W cm^−2^ could cause damage to normal tissues. The sample was irradiated by a laser with 1.5 W cm^−2^ for 10 min and the temperature can increase to 60 ºC, which could effectively kill cancer cells, but no damage to normal tissues, thus an 808 laser with 1.5 W cm^−2^ could be chose for the following experiments.

It can be seen from Fig. 4D, E that the temperature change increased with increasing concentration of AuNR@FA-PR/PEG/CDDP solution and the extension of the irradiation time. The solution with various concentration (0, 25, 50, 100, 200 µg mL^−1^) was irradiated with an 808 nm laser (1 W cm^−2^) for 10 min, the temperature increased 7, 21, 26, 34 and 42 °C, respectively, and the final temperature reached to 33, 47, 52, 60 and 68 °C.

It is also quite important to evaluate the photothermal stability of AuNR@FA-PR/PEG/CDDP for further clinical application (Fig. 4H). The maximum temperature rose above 69 °C during four LASER ON/OFF cycles with an 808 nm laser irradiation, that demonstrated the AuNR@FA-PR/PEG/CDDP had excellent stability and could be applied in the clinic in the future. The photothermal conversion ability was investigated, too. Firstly, the heat transfer time constant (τs) of the system was determined by the linear time data from the cooling period (Additional file 1: Fig. S3), and the photothermal efficiency was 26%.

In order to further study the relationship between temperature and irradiation intensity/material concentration, a real-time infrared thermal imaging experiment of AuNR@FA-PR/PEG/CDDP was implemented (Fig. 4F,G). When AuNR@FA-PR/PEG/CDDP with predetermined concentration (50 μg mL^−1^) was irradiated with various irradiation power intensities, the real-time infrared thermal imaging signal enhanced with irradiation intensity increasing, which confirmed the temperature elevated with irradiation intensity increasing and the irradiation time prolong. The Fig. 4G showed the real-time infrared thermal imaging signal rose with the concentration increasing of AuNR@FA-PR/PEG/CDDP, proving temperature elevating with the increased concentration of AuNR@FA-PR/PEG/CDDP. All photothermal performance results confirmed that AuNR@FA-PR/PEG was a promising nanocomposite for PTT.

### In vivo photothermal effect

**Fig. 5:**
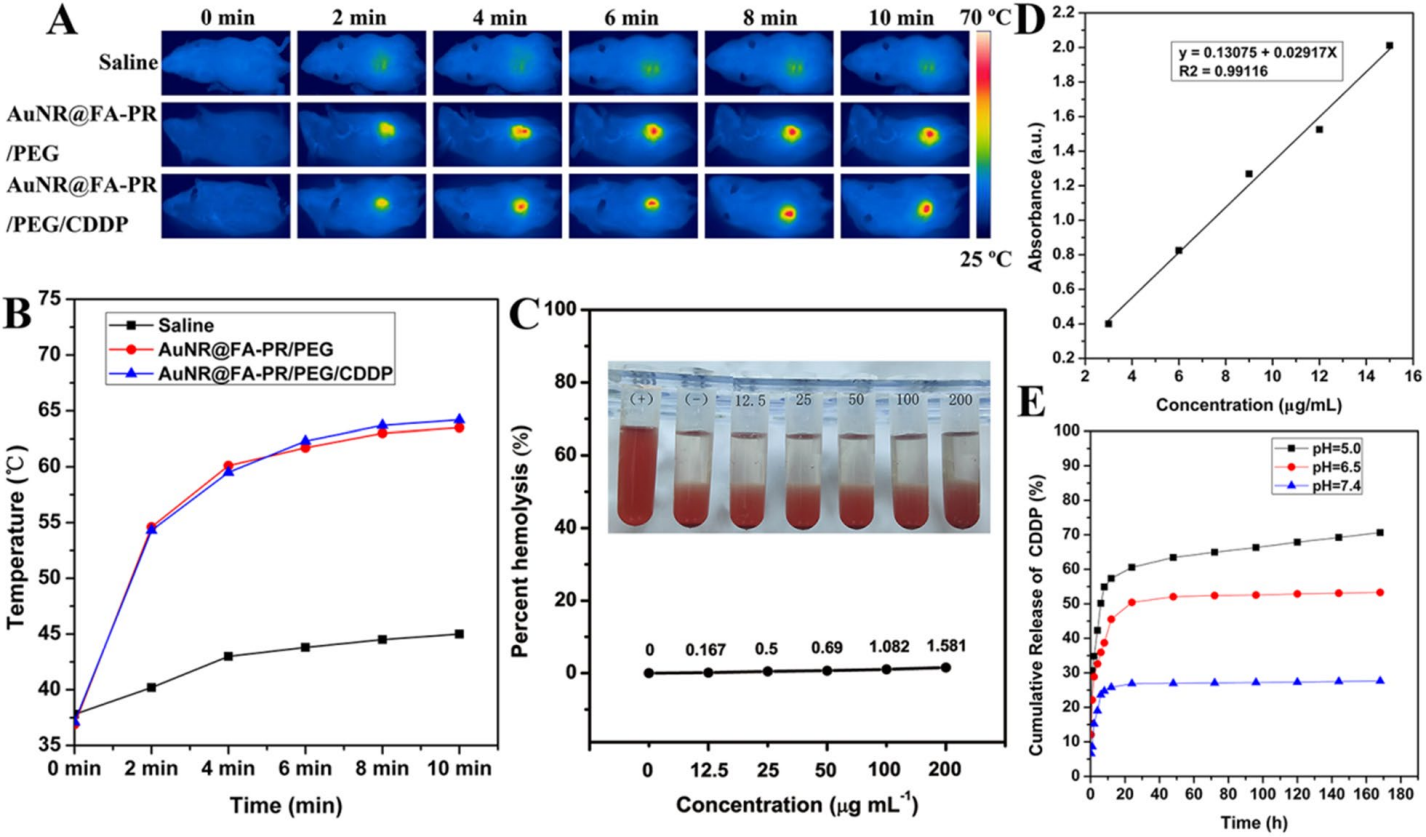
**A** Thermal camera images of tumor-bearing mice and **B** temperature curve of tumor areas at 6 h intravenous injection with Saline, AuNR@FA-PR/PEG and AuNR@FA-PR/PEG/CDDP; **C** Hemolysis percentage of RBC at various concentrations of AuNR@FA-PR/PEG/CDDP; **D** The standard curve of CDDP; **E** The drug release profiles of AuNR@FA-PR/PEG/CDDP at different pH values

In order to evaluate the in vivo photothermal effect of AuNR@FA-PR/PEG/CDDP, H22 tumor-bearing Kunming mice models were established. Saline, AuNR@FA-PR/PEG and AuNR@FA-PR/PEG/CDDP were injected into tumor-bearing mice via the tail vein, respectively. The saline group served as a blank control group. The tumor areas of three treatment group mice were irradiated by an 808 nm laser at power intensity of 1.0 W cm^−2^ for 10 min. The real-time infrared thermal imaging signals of tumors were recorded on an infrared thermal imaging camera, and the tumor temperatures of treatment mice were measured, the results were displayed in Fig. 5A, B. It could be found that the real-time infrared thermal imaging signals of tumors were weak in saline treatment group, and the tumor temperature changed little with the prolonged irradiating time, the temperature of tumor area only rose to 45 °C with irradiating for 10 min. The tumor areas infrared thermal imaging signal of AuNR@FA-PR/PEG and AuNR@FA-PR/PEG/CDDP treatment group mice became strong with prolonged irradiating time, and the tumor temperatures changed large, that could reach up to 63.5 °C and 64.2 °C, respectively. The results confirmed that rod-like AuNR@FA-PR/PEG/CDDP had an excellent photothermal performace in vivo comparing with spherical gold nanomaterials [28] and should be a promising combination therapy nanomaterial.

### Drug loading of AuNR@FA-PR/PEG and in vitro simulated release

The CDDP was loaded in nanocomposites through the chelation bonds between the rich carboxyl group in AuNR@FA-PR/PEG and cisplatin. The drug loading content was calculated by standard curve method. First, a standard curve equation was established, and the equation is: y = 0.13075x + 0.02917. The AuNR@FA-PR/PEG/CDDP was prepared into a solution of 200 µg mL^−1^, the content of CDDP was detected according to the above standard curve equation (Fig. 5D), and the drug loading can reach 7.4%.

In order to study the sustained release behavior of CDDP loaded in AuNR@FA-PR/PEG/CDDP in vitro, the in vitro release experiment was carried out in three different pH (5.0, 6.5, 7.4) values media at 37 °C for 168 h. Obviously, the release trend of CDDP loaded in AuNR@FA-PR/PEG treated with three pH value media all became gentle with the prolonged time (Fig. 5E). The release amount of CDDP was only 27.6% in pH 7.4 release media at 168 h, while it could reach up to 53.3% and 70.7% in pH 6.5 and 5.0 media, respectively. The release amount under acidic conditions were much higher than that in the PBS buffer solution with pH 7.4, which confirmed the CDDP-loaded in AuNR@FA-PR/PEG was pH-responsive. The loaded drug was stable under neutral environment (pH 7.4), and could be released in the acidic environment of tumor. The reason for this may be that the Pt–O bond is unstable in acidic environments and relatively easy to hydrolyze and break. The microenvironment of the tumors is acidic, while physiological pH is close to neutral, therefore the CDDP-loaded in AuNR@FA-PR/PEG can be successfully released in tumor microenvironment for effective cancer treatment, and stable in normal tissue and physiological environment, that can reduce the toxic side effects of the antitumor drug toward organism and improve the anticancer effect.

To investigate the effect of photothermal on the release of CDDP-loaded in AuNR@FA-PR/PEG, the drug release experiments of AuNR@FA-PR/PEG/CDDP under an 808 nm laser irradiation at three pH values (7.4, 6.5, 5.0) were carried out, and the results were shown in Additional file 1: Figure S4. The results displayed that the release profiles of AuNR@FA-PR/PEG/CDDP at pH 7.4 value with and without laser irradiation were almost the same. While the drug amounts of CDDP with laser irradiation at pH 6.5 and 5.0 were 63% and 85.6%, respectively, which are higher than these (53.3%, 70.7%) of CDDP without laser irradiation at the same pH (6.5, 5.0). All the results proved that the photothermal could promote the release of CDDP-loaded in AuNR@FA-PR/PEG, and photothermal therapy and chemotherapy will exhibit excellent synergistic antitumor effect.

### Hemolysis experiment

The hemolysis is caused by the non-specific interaction of nanomaterials with hemoglobin, which can seriously affect practical application of nanomaterials in the nanomedicine field and must be overcome. Thus, the blood compatibility of AuNR@FA-PR/PEG/CDDP should be considered and the hemolysis experiment of AuNR@FA-PR/PEG/CDDP was carried out. The distilled water was used as a positive control group, and the saline group was used as a negative control group. The result was displayed in Fig. 5C, it can be seen from the figure that the positive control group caused hemolysis, while the negative control group did not appear hemolysis phenomenon. The hemolysis rate of AuNR@FA-PR/PEG/CDDP increased with the increasing concentration at a certain concentration range (from 12.5 to 200 µg mL^−1^), while the hemolysis rate is only 1.581 at the concentration up to 200 µg mL^−1^, which is much less than 5%. The result confirmed that AuNR@FA-PR/PEG/CDDP had excellent blood compatibility and met the standard medical requirements. Thus, AuNR@FA-PR/PEG/CDDP can be used as nanomedicine for cancer treatment.

### In vitro cytotoxicity assay and photothermal cytotoxicity assay of AuNR@FA-PR/PEG and AuNR@FA-PR/PEG/CDDP

**Fig. 6:**
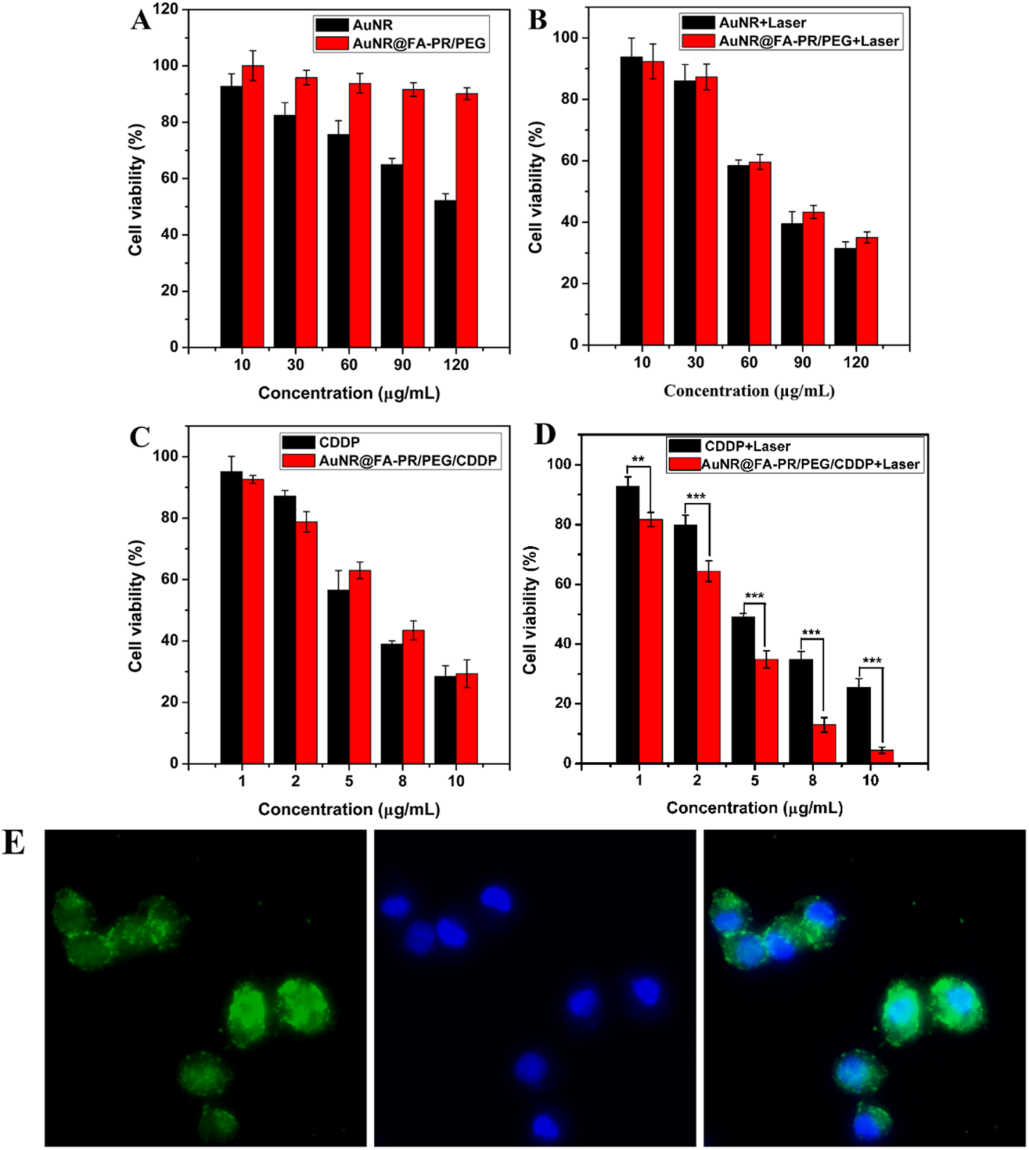
**A** The cytotoxicity of AuNR and AuNR@FA-PR/PEG to HepG2 cells; **B** The cytotoxicity of AuNR and AuNR@FA-PR/PEG to HepG2 cells irradiated with an 808 nm laser (1 W cm^−2^); **C** The cytotoxicity of CDDP and AuNR@FA-PR/PEG/CDDP to HepG2 cells; **D** The photothermal cytotoxicity of CDDP and AuNR@FA-PR/PEG/CDDP to HepG2 cells irradiated with an 808 nm laser (1 W cm.^−2^); **E** The cellular uptake of AuNR@FA-PR/PEG/CDDP by HepG2 cells. Statistical significance: **P < 0.01; ***P ≤ 0.001

The in vitro cytotoxicity and photothermal cytotoxicity of AuNR@FA-PR/PEG and AuNR@FA-PR/PEG/CDDP were evaluated with HepG2 cells by the 3-(4,5-dimethyl-2-thiazolyl)-2,5-diphenyl-2-H-tetrazolium (MTT) assay, and the results were shown in Fig. 6. The cytotoxicity of AuNRs increased with the increasing concentration, and the cell activity dropped to 50% with 120 µg mL^−1^ of AuNRs treatment. While the AuNR@FA-PR/PEG showed excellent biocompatibility to HepG2 cells, the cells activity still remained over 90% with 120 µg mL^−1^ of AuNR@FA-PR/PEG treatment, the reason is that FA-PR and PEG in surface of AuNR@FA-PR/PEG are biocompatible. The cells treated with AuNRs and AuNR@FA-PR/PEG were irradiated by an 808 nm laser at power intensity of 1.5 W cm^−2^, the results showed that the cell activities decreased with the increasing concentration, the tumor suppression rate of two materials with maximum concentration were 47.8% and 68.5%, respectively, which confirmed the tumor suppression effect of AuNRs was better than that of AuNR@FA-PR/PEG, it is because that the Au amount in AuNRs is higher than that in the same concentration AuNR@FA-PR/PEG.

In order to investigate the synergistic anti-tumor effect of chemotherapy and photothermal therapy, the cytotoxicity experiment of CDDP and AuNR@FA-PR/PEG/CDDP toward HepG2 were carried out, as shown in Fig. 6C, D. The results displayed that the cytotoxicity of AuNR@FA-PR/PEG/CDDP toward HepG2 gradually increased with the concentration of AuNR@FA-PR/PEG/CDDP solution, the activity of HepG2 cells treated by AuNR@FA-PR/PEG/CDDP solution with a drug concentration of 10 µg mL^−1^ is 29.3%, basically as same as that of CDDP, which confirmed the antitumor drug could be successfully released continuously and maintained original efficacy. Figure 6D showed the cytotoxicity results of CDDP and AuNR@FA-PR/PEG/CDDP toward HepG2 cells under irradiating by an 808 nm laser with power intensity of 1.0 W cm^−2^, appearing that the cytotoxicities of CDDP to HepG2 cells with and without irradiating by an 808 nm laser were almost the same. While the cytotoxicity of AuNR@FA-PR/PEG/CDDP toward HepG2 cells under laser irradiation was much higher than that without laser irradiation, the survival rate of HepG2 cells is only about 4.4%, which confirmed AuNR@FA-PR/PEG/CDDP had an excellent photothermal treatment effect. Comparing CDDP with irradiating and AuNR@FA-PR/PEG/CDDP with irradiating treatment groups, the later exhibited significant cytotoxicity, and there were significant differences in cytotoxicity between the two treatment groups, all results showed that the drug and photothermal therapy can effectively treat cancer together.

To investigate the biosafety of photothermal and chemotherapy, the cytotoxicity assay and photothermal cytotoxicity assays of CDDP and AuNR@FA-PR/PEG/CDDP with and without an 808 nm laser irradiation to human normal liver cells HL-7702 were carried out, and the results were showed in Additional file 1: Fig.S5. It can be seen from the figure that the cytotoxicity increased with the increasing concentration of CDDP in CDDP and CDDP+Laser treatment groups, and the photothermal has a slight effect on the cytotoxicity of CDDP. While the cell activity in AuNR@FA-PR/PEG/CDDP with and without an 808 nm laser irradiation treatment groups only appeared a slight decrease with increasing concentration of AuNR@FA-PR/PEG/CDDP, which may be the reason of the trace amounts of CDDP released from AuNR@FA-PR/PEG/CDDP. All results proved the biosafety of photothermal and chemotherapy.

### Cellular uptake experiment of AuNR@FA-PR/PEG/CDDP 

AuNR@FA-PR/PEG/CDDP was labeled with fluorescein isothiocyanate (FITC), and then the FITC-labeled AuNR@FA-PR/PEG/CDDP was co-cultured with HepG2 cells, finally, the cellular uptake of AuNR@FA-PR/PEG/CDDP by HepG2 cells was investigated with a confocal laser scanning microscopy, the result was shown in Fig. 6E. The result displayed that AuNR@FA-PR/PEG/CDDP could successfully enter into the cytoplasm of HepG2 cells, which confirmed that AuNR@FA-PR/PEG/CDDP can be internalized by HepG2 cells through cellular endocytosis.

### In vivo real-time imaging

**Fig. 7:**
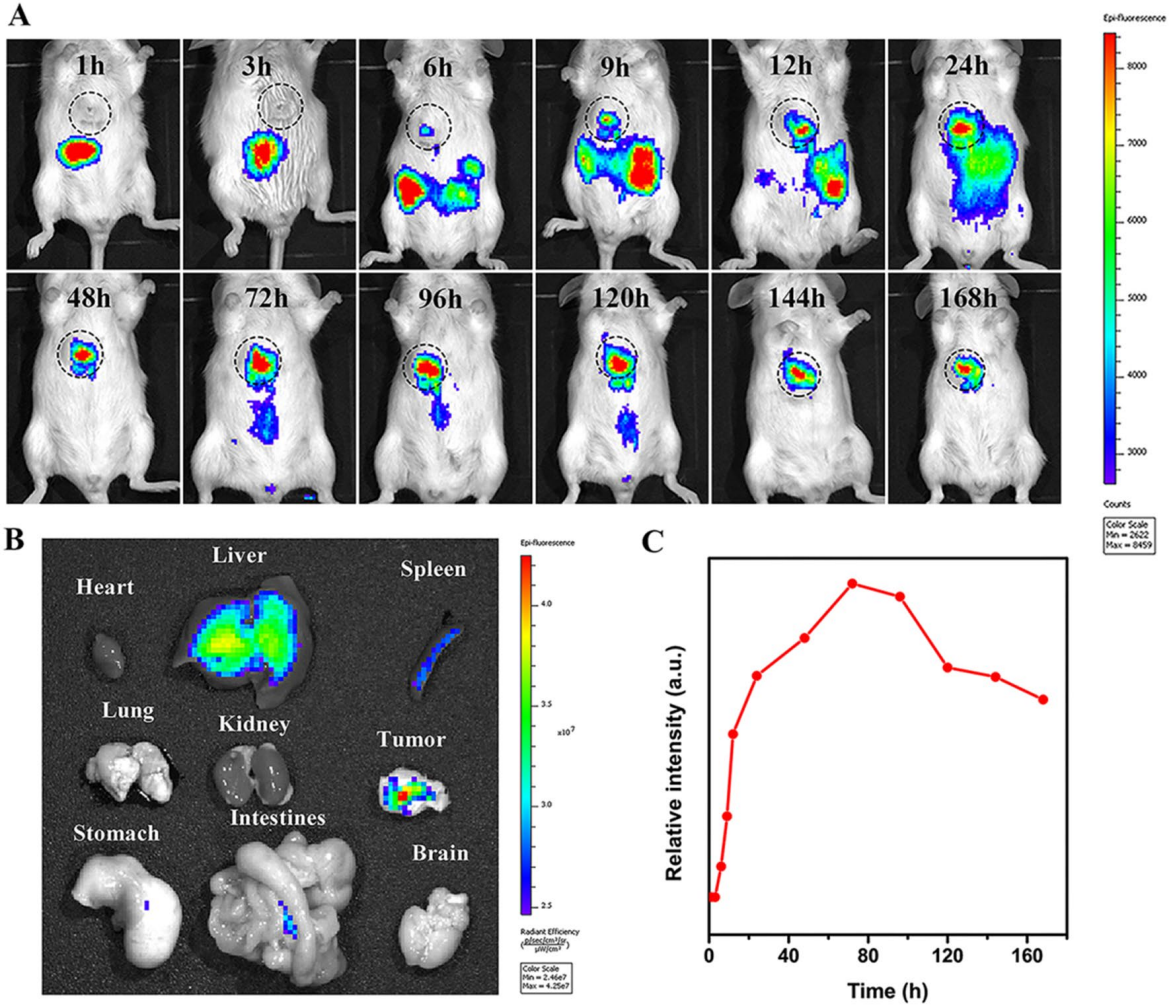
**A** In vivo fluorescence imaging; **B** The fluorescence image of various organs at 168 h after intravenous injection; **C** relative fluorescence intensity of tumor in tumor-bearing mice after injecting with NIR-797-labeled AuNR@FA-PR/PEG/CDDP intravenously at different time points

To investigate the biological distribution of AuNR@FA-PR/PEG/CDDP in vivo and the accumulation and retention in tumor, the in vivo real-time imaging experiment of NIR797-labeled AuNR@FA-PR/PEG/CDDP was carried out with a MaestroTM in vivo imaging system, and the results were displayed in Fig. 7. It could be seen from the Fig. 7A that the tumor area appeared weak fluorescence signal at 6 h after intravenous injection, which became strong over time, and at 168 h after intravenous injection, the tumor area still appeared strong fluorescence signal. The results proved that AuNR@FA-PR/PEG/CDDP could quickly enter into tumors, enrich and stay for a long time. On the contrary, the liver and intestine regions showed strong fluorescence signal at 1 to 9 h following intravenous injection, while the fluorescence signal became weak over time, the results confirmed that AuNR@FA-PR/PEG/CDDP could be excreted through liver and intestine metabolism. The ROI function of IVIS Lumina XRMS Series was used to quantity the fluorescence signal of the tumor region at different time points after tail vein injection, and the quantitative results were shown in Fig. 7C. The results displayed that the fluorescence intensity in the tumor region gradually augmented with extending time, and it reached up to a maximum at 72 h after injecting via tail vein, which indicated the active accumulation of AuNR@FA-PR/PEG/CDDP in the tumor area though the folate receptor-mediated endocytosis. The fluorescence intensity in the tumor region gradually fell down over time, however, which was still strong at 168 h following intravenous injection, all results proved that AuNR@FA-PR/PEG/CDDP could quickly accumulate and delay for a long time in tumor area, and the decay rate of the fluorescence intensity in tumor area was slow.

Further, at 168 h after intravenous injection, the mice treated with NIR-797-labeled AuNR@FA-PR/PEG/CDDP was sacrificed, and then the tumor and major organs including heart, liver, spleen, lung, kidney, intestine, stomach and brain were excised and subjected to fluorescence imaging, the results were shown in Fig. 7B. Weak fluorescence signal was detected in liver, spleen and intestines, and no fluorescence signals were detected in other normal tissues, which proved AuNR@FA-PR/PEG/CDDP could be excreted through hepatointestinal metabolism. While strong fluorescence signal could be detected in tumor area, indicating that AuNR@FA-PR/PEG/CDDP could enrich and keep for a long time in tumor region. Therefore, AuNR@FA-PR/PEG/CDDP is suitable as a nanomedicine for the treatment of cancer.

### The accumulation of AuNR@FA-PR/PEG/CDDP in tumor at different times

In order to study the accumulation of AuNR@FA-PR/PEG/CDDP in tumor, the real-time imaging and quantification experiments were carried out, and the results were provided in the supporting information (Additional file 1: Fig. S6 and S7). Each group mice were imaged before sacrificing and the results showed in Additional file 1: Fig. Additional file 1: S6A. 6 h after tail vein injection, sample entered into tumor, and the fluorescence intensity in tumor became strong over time. The fluorescence signal reached the strongest at 8 h after tail vein injection, and became weak over time. But the fluorescence signal decays very slowly, which was still strong 96 h after tail veil injection. Additional file 1: Fig. S6B showed the quantitative results. The mice in each group were dissected and the tumors were collected for fluorescence imaging after in vivo imaging, and the fluorescence at the tumor site was quantified. The results showed that the fluorescence signal appeared in the tumor area 6 h after intravenous injection, became strong with time, and reached the strongest at 48 h after intravenous injection, and then slowly decayed with time, which proved that AuNR@FA-PR/PEG/CDDP could quickly accumulate and stay for a long time in tumor.

In addition, the content of Au in tumors was detected to study the enrichment of AuNR@FA-PR/PEG/CDDP in tumors over time, and the results showed in Additional file 1: Fig. S7. The results were consistent with those of in vivo imaging. All results proved that AuNR@FA-PR/PEG/CDDP could easily enter tumor, accumulate and stay for a long time in tumor. Therefore, AuNR@FA-PR/PEG/CDDP will have excellent antitumor effect.

### In vivo antitumor efficacy

**Fig. 8:**
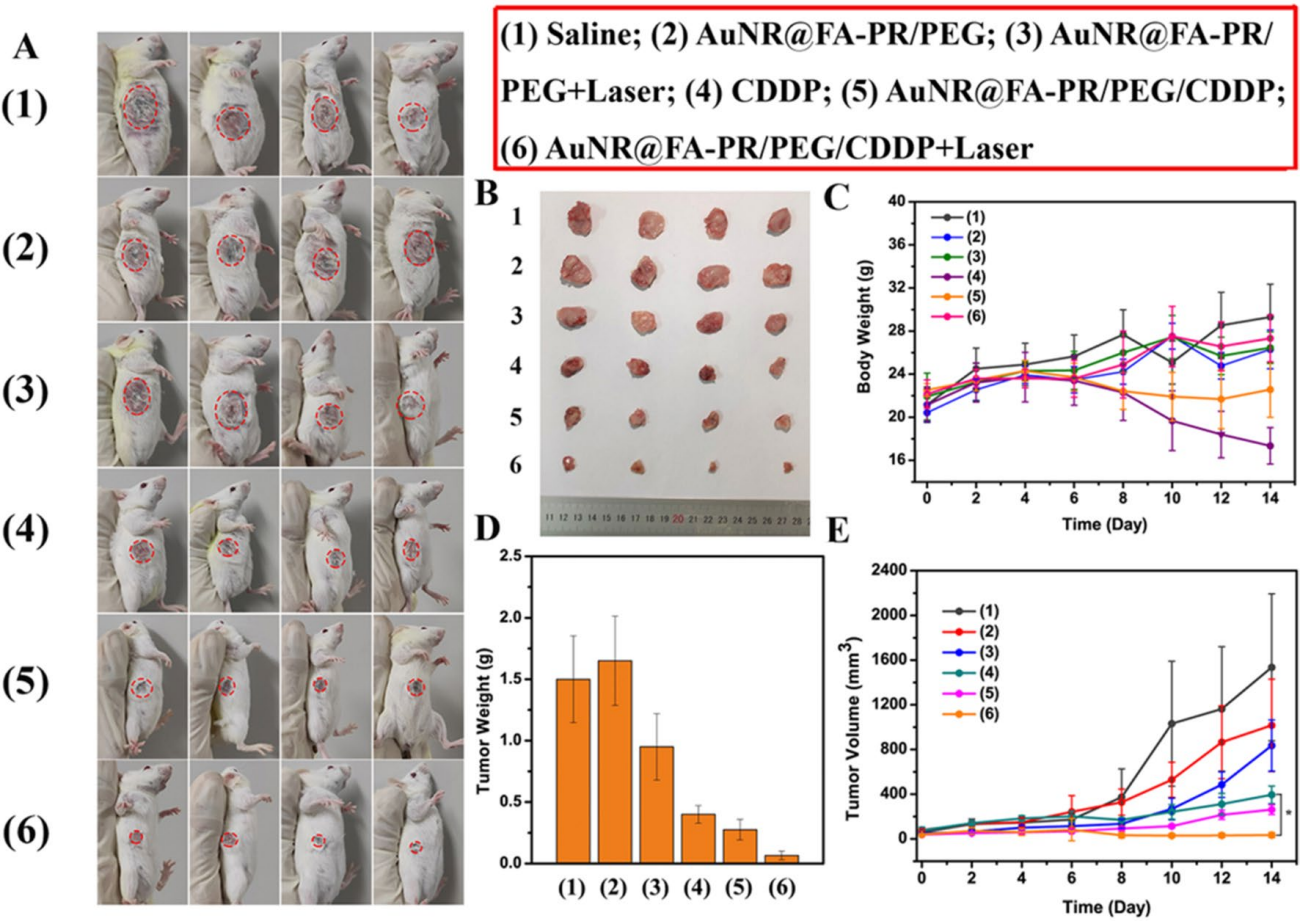
**A** Representative pictures for H22 tumor-bearing mice at 14th day of treatment with saline, AuNRs@FA-PR/PEG, AuNR@FA-PR/PEG+Laser, CDDP, AuNR@FA-PR/PEG/CDDP, AuNR@FA-PR/PEG/CDDP+Laser; **B** Images of tumors in each group taken out from the sacrificed H22 tumor-bearing mice at the end point of research; **C** Weight changes in each group of mice during the treatments;**D** Representative pictures for tumors excised from the mice with different treatments after the 14 day treatment; **E** Tumor volumes changes in each group of mice during the treatments (*P < 0.05); (1) Saline; (2) AuNR@FA-PR/PEG; (3) AuNR@FA-PR/PEG+Laser; (4) CDDP; (5) AuNR@FA-PR/PEG/CDDP; (6)AuNR@FA-PR/PEG/CDDP + Laser; (808 nm, 1 W cm^−^.^2^, 6 min)

The in vivo combined anti-tumor activity of chemotherapy and photothermal therapy of AuNR@FA-PR/PEG/CDDP by a laser irradiation was evaluated by using H22 tumor-bearing Kunming mice as model animals, and the results were shown in Fig. 8. Figure 8A displayed the photos of tumor-bearing mice at 14th day treated with six samples (saline, AuNR@FA-PR/PEG, AuNR@FA-PR/PEG+Laser, CDDP, AuNR@FA-PR/PEG/CDDP, AuNR@FA-PR/PEG/CDDP+Laser). It can be seen from the figure that the tumors of mice treated with saline and AuNR@FA-PR/PEG appeared the biggest, those in AuNR@FA-PR/PEG+Laser, CDDP and AuNR@FA-PR/PEG/CDDP treatment groups were smaller than that in above two groups, which proved AuNR@FA-PR/PEG+Laser, CDDP and AuNR@FA-PR/PEG/CDDP had a certain anti-tumor effect. Comparing with other groups, the tumors in the AuNR@FA-PR/PEG/CDDP+Laser treatment group were the smallest, which confirmed the synergistic treatment of chemotherapy and photothermal therapy had a significant therapy effect for cancers. The tumors were excised from mice at the 14th day after treatment with saline, AuNR@FA-PR/PEG, AuNR@FA-PR/PEG+Laser, CDDP, AuNR@FA-PR/PEG/CDDP, AuNR@FA-PR/PEG/CDDP+Laser, respectively, the photos of tumors and the tumor weight measurements were showed in Fig. 8B, D, the results also demonstrated AuNR@FA-PR/PEG/CDDP+Laser displayed excellent anti-tumor effect.

After therapy, the weight of mice and the volume of tumors were measured every other day until the 14th day. As presented in Fig. 8C, E, the body weight of mice in CDDP treatment group dropped significantly, which proved the biological toxicity of CDDP. The body weights of mice in all other treatment groups showed no significant loss, certifying the biosafety of materials, drug-loaded materials and drug-loaded material with laser irradiation. The tumor volume results displayed that saline and AuNR@FA-PR/PEG showed no any anti-tumor effect. Comparing with above two groups, AuNR@FA-PR/PEG+Laser, CDDP and AuNR@FA-PR/PEG/CDDP treatment groups appeared a certain therapy effect for tumor. The AuNR@FA-PR/PEG/CDDP+Laser treatment group showed the most excellent anti-tumor effect, which confirmed AuNR@FA-PR/PEG/CDDP should be a promising drug-loaded material used in the synergy therapy of chemotherapy and photothermal therapy for cancers.

### Determination of cell apoptosis and proliferation and histology studies

**Fig. 9:**
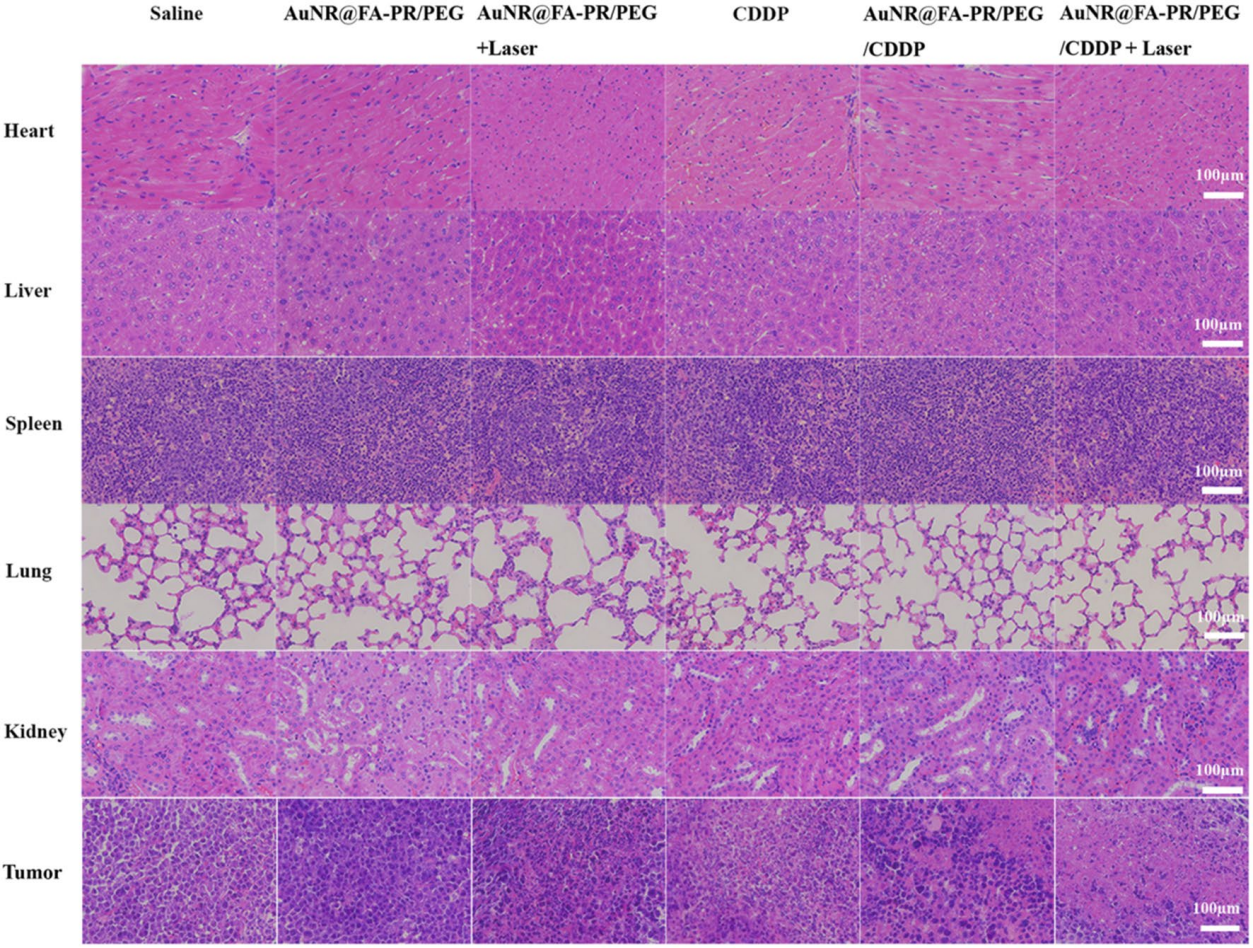
H&E staining of the major organs and tumors after 14 days treatment (Saline; AuNR@FA-PR/PEG; AuNR@FA-PR/PEG+Laser; CDDP; AuNR@FA-PR/PEG/CDDP; AuNR@FA-PR/PEG/CDDP + Laser) (808 nm, 1.0 W cm.^−2^, 6 min) (Scale bars are 100 μm)

**Fig. 10:**
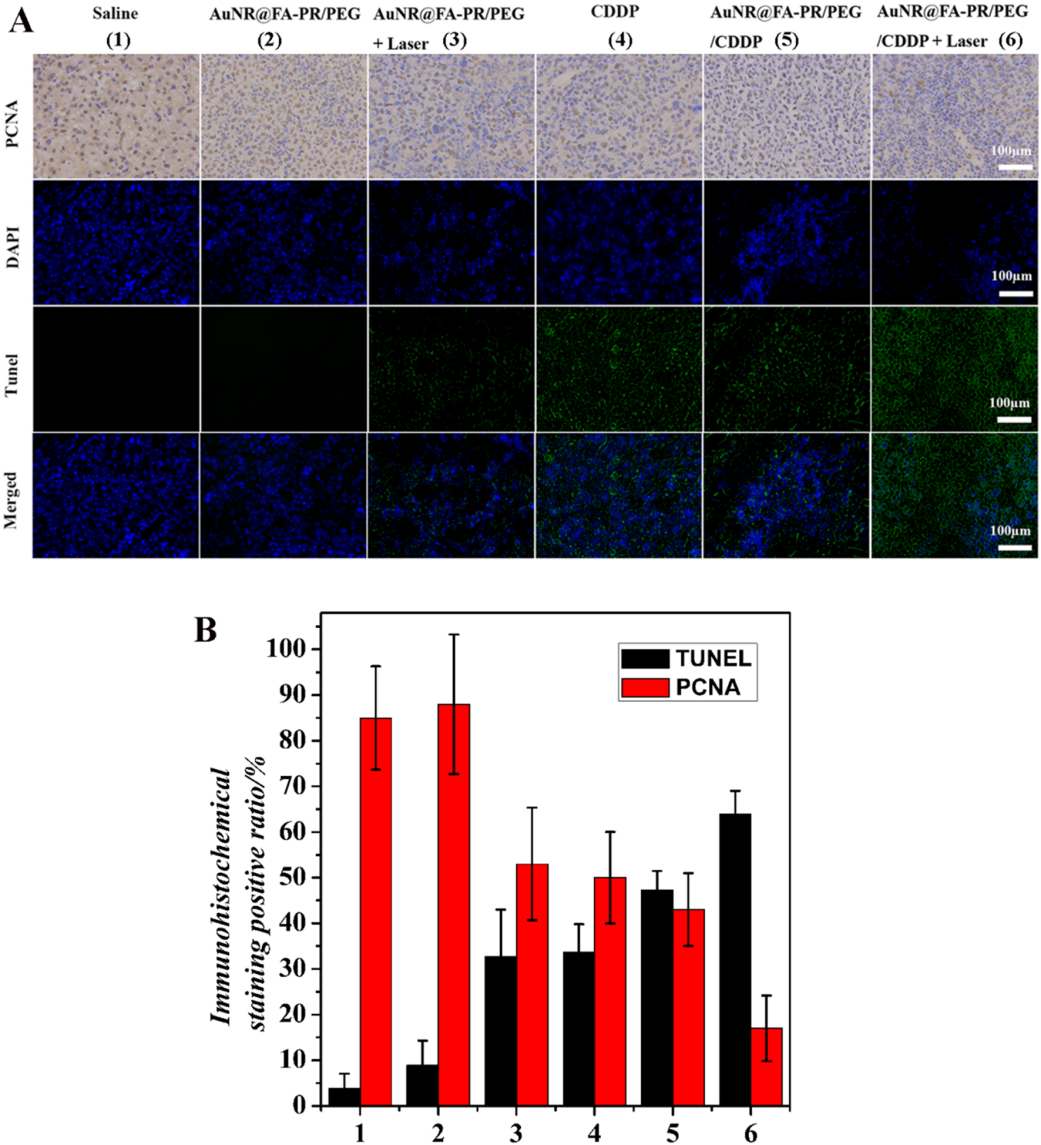
**A** PCNA staining and Tunel staining of tumor tissue after 14 days of treatment (Saline; AuNR@FA-PR/PEG; AuNR@FA-PR/PEG+Laser; CDDP; AuNR@FA-PR/PEG/CDDP; AuNR@FA-PR/PEG/CDDP+Laser) (808 nm, 1.0 W cm.^−2^, 6 min)(scale bars are 100 μm); **B** Quantification (*P < 0.01) of TUNEL and PCNA expression

To further evaluate the biosafety and combined anti-tumor effect of AuNR@FA-PR/PEG/CDDP, the H&E, TUNEL and PCNA assays were carried out, and the results were showed in Figs. 9 and 10. The heart, liver, spleen, lung and kidney of mice in all materials treatment groups showed no lesions, the result demonstrated that AuNR@FA-PR/PEG/CDDP+Laser had excellent biosafety. The H&E assay results of tumors from mice treated with various samples displayed that AuNR@FA-PR/PEG/CDDP+Laser showed the best antitumor effect.

To verify the effect of AuNR@FA-PR/PEG/CDDP on normal tissues and cells at an 808 nm laser, the H&E assays of tumor and nearby tissues were carried out, and the result was showed in Additional file 1: Fig. S8. The part outlined by the yellow dotted line is the tumor damage area. It can be seen from the figure that the tumor and nearby tissues did not appear significant damage. Different degrees of damage appeared in the tumor areas of the other treatment groups, especially a large tumor area appeared damage in the AuNR@FA-PR/PEG/CDDP+Laser treatment group, while there is slight damage to its peripheral cells. All results demonstrate the biosafety of photothermal and chemotherapy-mediated AuNR@FA-PR/PEG/CDDP.

The PCNA results showed that the saline, materials and single treatment groups had many brown cells, indicating the cell proliferation. While the drug-loaded material with laser irradiation appeared a few brown cells, which demonstrated AuNR@FA-PR/PEG/CDDP+Laser had an excellent therapy effect for cancer. The tumor apoptosis was further examined though terminal deoxynucleotidyl transferase-mediated dUTP-biotin nick end labeling (TUNEL) assays. As shown in Fig. 10, comparing with other treatment groups, significant apoptosis cells were observed in the AuNR@FA-PR/PEG/CDDP+Laser treatment group, indicating an excellent synergy therapy effect of chemotherapy and photothermal therapy for cancers. Quantitative analysis results of PCNA and TUNEL assays were also shown in Fig. 10B. In the case of PCNA assay that determines the proliferation active cells, high proliferation levels are observed in saline and AuNR@FA-PR/PEG treatment groups. Comparing to the AuNR@FA-PR/PEG+Laser, CDDP and AuNR@FA-PR/PEG/CDDP-treated group, the percentage of PCNA-positive cells significantly decreases in AuNR@FA-PR/PEG/CDDP+Laser treatment group. Furthermore, the percentage of apoptosis cells determined by TUNEL assay in tumors treated by AuNR@FA-PR/PEG/CDDP+Laser is significantly higher than that in tumors treated by AuNR@FA-PR/PEG+Laser, CDDP and AuNR@FA-PR/PEG/CDDP. AuNR@FA-PR/PEG treatment group does not cause significant difference in apoptosis level when compared to saline treatment group. All results of PCNA and TUNEL assays were consistent with the in vivo therapeutic outcome.

## Conclusions

In this study, a rod-like hybrid nanosystem (AuNR@FA-PR/PEG/CDDP) forming from FA-PR and PEG modifying AuNR was successfully prepared for the combination of chemotherapy and photothermal therapy for cancer therapy. The hybrid nanosystem had excellent biocompatibility, enhanced cell membrane interaction, tumor targeting and pH-responsive drug release function. The TEM demonstrated the morphology of AuNR@FA-PR/PEG/CDDP was rod-like, and the length and aspect ratio of AuNR@FA-PR/PEG/CDDP were about 70 nm and 3.5, respectively. CDDP was loaded in the AuNR@FA-PR/PEG through the coordination bond with pH responsiveness, and could be released in acid conditions. The photothermal performance of AuNR@FA-PR/PEG/CDDP was studied through in vitro and in vivo experiments, the results proved the excellent photothermal properties of AuNR@FA-PR/PEG/CDDP. The synergistic effects of AuNR@FA-PR/PEG/CDDP with chemotherapy and photothermal therapy were also evaluated through in vitro and in vivo assays. The in vivo real-time imaging assay confirmed that AuNR@FA-PR/PEG/CDDP could quickly enter and accumulate in the tumor because of active targeting and rod-shape morphology, and stay in tumor for a long time. The in vitro and in vivo experiments also demonstrated that AuNR@FA-PR/PEG/CDDP had significant synergistic effects of chemotherapy and photothermal therapy for cancer treatment. Therefore, AuNR@FA-PR/PEG/CDDP will be a promising hybrid nanosystem for dual-modal combination therapy for cancer therapy.

## Supplementary Information

**Additional file 1: **The Supporting Information is available free of charge at Reagents, materials and characterization; instruments; experimental procedures; ^1^H NMR spectra of FA-PEG-SH, ^1^H NMR characterization of α-CD and α-CD-COOH;The photothermal and natural cooling curve of AuNR@FA-PR/PEG/CDDP; The drug release profiles of AuNR@FA-PR/PEG/CDDP with an 808 nm laser (1.5 W cm^-2^) at different pH values; The cytotoxicity of CDDP and AuNR@FA-PR/PEG/CDDP to HL-7702 cells irradiated with and without an 808 nm laser (1 W cm^-2^); In vivo fluorescence imaging and the fluorescence intensity of dissected tumor at different time points; AuNR@FA-PR/PEG/CDDP content in tumors at different time points; H&E staining of the tumor and nearby tissues.

## Data Availability

All data generated or analyzed during this study are included in this published article and the Supporting Information.
