# Supplementary material for: Rod-like hybrid nanomaterial with tumor targeting and pH-responsive for cancer chemo/photothermal synergistic therapy
Source: J Nanobiotechnology. 2022 Jul 16;20:332. doi: 10.1186/s12951-022-01527-1 (PMC9287864; doi:10.1186/s12951-022-01527-1)
Supplement: Supplementary file 1 — Additional file 1: The Supporting Information is available free of charge at Reagents, materials and characterization; instruments; experimental procedures; 1H NMR spectra of FA-PEG-SH, 1H NMR characterization of α-CD and α-CD-COOH;The photothermal and natural cooling curve of AuNR@FA-PR/PEG/CDDP; The drug release profiles of AuNR@FA-PR/PEG/CDDP with an 808 nm laser (1.5 W cm-2) at different pH values; The cytotoxicity of CDDP and AuNR@FA-PR/PEG/CDDP to HL-7702 cells irradiated with and without an 808 nm laser (1 W cm-2); In vivo fluorescence imaging and the fluorescence intensity of dissected tumor at different time points; AuNR@FA-PR/PEG/CDDP content in tumors at different time points; H&E staining of the tumor and nearby tissues. [file 12951_2022_1527_MOESM1_ESM.docx]

**Additional file Information**

**Rod-Like Hybrid Nanomaterial with Tumor Targeting and pH-Responsive for Cancer Chemo/Photothermal Synergistic Therapy**

Shaochen Wang^#1^, Qiaoqiao Zhou^#1^, Shuling Yu^*1^, Shuang Zhao^1^, Jiahua Shi^*1^ and Jintao Yuan^*2^

1. Key Laboratory of Natural Medicine and Immune-Engineering of Henan Province, Henan University, Kaifeng, Henan, 475004, People’s Republic of China.

2. College of Public Health, Zhengzhou University, Zhengzhou, 450001, People’s Republic of China.

# Co-first Author

*Corresponding authors: yushuling@henu.edu.cn, sjiahua@henu.edu.cn, [jtyuan@zzu.edu.cn](mailto:jtyuan@zzu.edu.cn).

**MATERIALS AND CHARACTERIZATIONS**

**Material**

α-cyclodextrin(α-CD) (98%, adamas-beta), folic acid (FA) (98%, adamas-beta), AgNO_3_, (AR, Beijing Chemical Factory), polyethylene glycol (PEG) 4000 (98%, adamas-beta), β-mercaptoethylamine (98%, Shanghai Jingchun Co., Ltd.), HAuCl_4_ (99%, Shanghai Chemical Reagent Co., Ltd). mPEG2000 (99%, TCI), N-(3-dimethylaminopropyl)-N’-ethylcarbodiimide hydrochloride (EDC·HCl)(99%, adamas-beta), N-hydroxysuccinimide (NHS)(99%, adamas-beta), NaBH_4_ (99%, Aladdin), sodium dodecyl oleate (NaOL, 98%, adamas-beta), cetyltrimethylammonium bromide(CTAB, 98%, adamas-beta), cisplatin (Pt) (Aladdin), dialysis bags (MWCO:3500, Shanghai Yuanye Biotechnology Co., Ltd.)(MWCO: 1000, McBride), NIR-797 isothiocyanate was purchased from Sigma-Aldrich, 1-(4,5-dimethylthiazol-2-yl)-3,5-diphenyl-formazan (MTT) (VWR). Fetal bovine serum (FBS) (Hangzhou Sijiqing Biological Engineering Materials Co., Ltd.), RPMI 1640(BOSTER USA), DMEM (Solarbio), Trypsin (Solarbio), 1,4-dioxane, dimethyl sulfoxide (DMSO), N,N-dimethylformamide (DMF), and ether were provided by Tianjin Fuyu Fine Chemical Co., Ltd.

**Characterization**

The materials synthesized in the work were characterized by nuclear magnetic resonance technology (NMR) with an AVANCE-400 NMR spectrometer (Bruker Co., USA) using CDCl_3_ or dimethyl sulfoxide (DMSO)-d_6_ as the solvent and with tetramethylsilane (TMS) as the internal standard. The morphology of the product was studied by high-resolution transmission electron microscopy (HRTEM) on a JEM-2100 electron microscope at 120 kV. UV-vis absorption spectroscopy was performed on a UV-9100A ultraviolet spectrophotometer (Shimadzu, Kyoto, Japan). The aspect ratio of prepared gold nanorods is about 3. The photothermal performance was investigated with an 808 nm NIR laser. The cells fluorescence imaging was performed on a fluorescence microscope (Leica, DMi8). In vivo and in vitro photothermal imaging of hybrid nanomaterials were studied on a photothermal camera.

**Preparation of gold nanorods (AuNRs)**

The preparation process of AuNR is mainly divided into the following two steps:

Preparation of gold seeds: 182.5 mg CTAB was added in 5.15 mL of distilled water, then the system was heated to 50 °C in a water bath to dissolve CTAB, and then HAuCl_4_ solution (10mg/mL, 52.6 µL,) was added into above system at 30 °C, finally, fresh sodium borohydride (0.01 M, 300 μL) was slowly added dropwise into the reaction system with vigorous stirring, the reaction continued for another 2 min and the color of the solution turned from yellow to brownish yellow. The seed solution was used after standing at 25 ° C for 30 min in a water bath.

The preparation of gold nanorod (AuNR): CTAB (900 mg) and NaOL (123.4 mg) were added to a flask containing 25 mL of distilled water, and the system was heated to 50 ºC to make the samples fully dissolve, then the system was cooled to room temperature and placed in a water bath at 30 ºC. AgNO_3_ solution (0.04 M, 1.2 mL) was added to the above system under stirring, and then 23.96 mL of distilled water was added to the reaction system and stirred evenly. HAuCl_4_ solution (10 mg/mL, 1 mL) was added to above solution and the system stood for 15 min, after that the solution was stirred 1.5 h with 700 rpm per min. 210 μL concentrated hydrochloric acid was added to the reaction solution and the speed of the stirrer was adjusted to 400 rpm, and then the system is allowed to stand for 15 min. To above reaction solution ascorbic acid (AA) solution (0.064 M, 125 μL) was added with stirring, then 80 mL of seed solution was added into the reaction solution with vigorous stirring, and then the system was stood for 12 h at 30 ºC. The mixture was centrifuged at a speed of 8500 rpm for 30 min, and the precipitation was collected and washed with deionized water for several times. Finally, the AuNRs were dispersed in 1 mL deionized water for further use.

**Preparation of carboxylated α-CD**

α-CD (10 g, 0.0103 mol) was dissolved in 30 ml of dehydrated DMSO, then DMAP (757.45 mg, 6.2 mmol) and succinic anhydride (6.2 g, 0.0620 mol ) were added, and the reaction was carried out under magnetic stirring for 24 h at room temperature. The above solution was slowly dropped into cold acetone to precipitate the product, the precipitate was collected though filtering and washed three times with acetone, and then the precipitate was placed in a vacuum drying oven for 3 days at room temperature. A white solid powder α-CD-COOH was obtained.

**Preparation of sulfhydryl mPEG (mPEG-SH)**

mPEG 2000 (4 g) and CDI (421mg) were dissolved in 20 mL of anhydrous DMF, the samples were dissolved by ultrasound. The reaction continued for 12 h under nitrogen protection conditions at room temperature. Then β-mercaptoethylamine (2.61 mmol, 201 mg) was added to the reaction system, and the reaction continued under the protection of N2 for 24 h, then the solution was dialyzed with 1 kDa dialysis bag against deionized water to remove the solvent and unreacted substances. Finally, the dialysate was lyophilized to give a white fluffy solid.


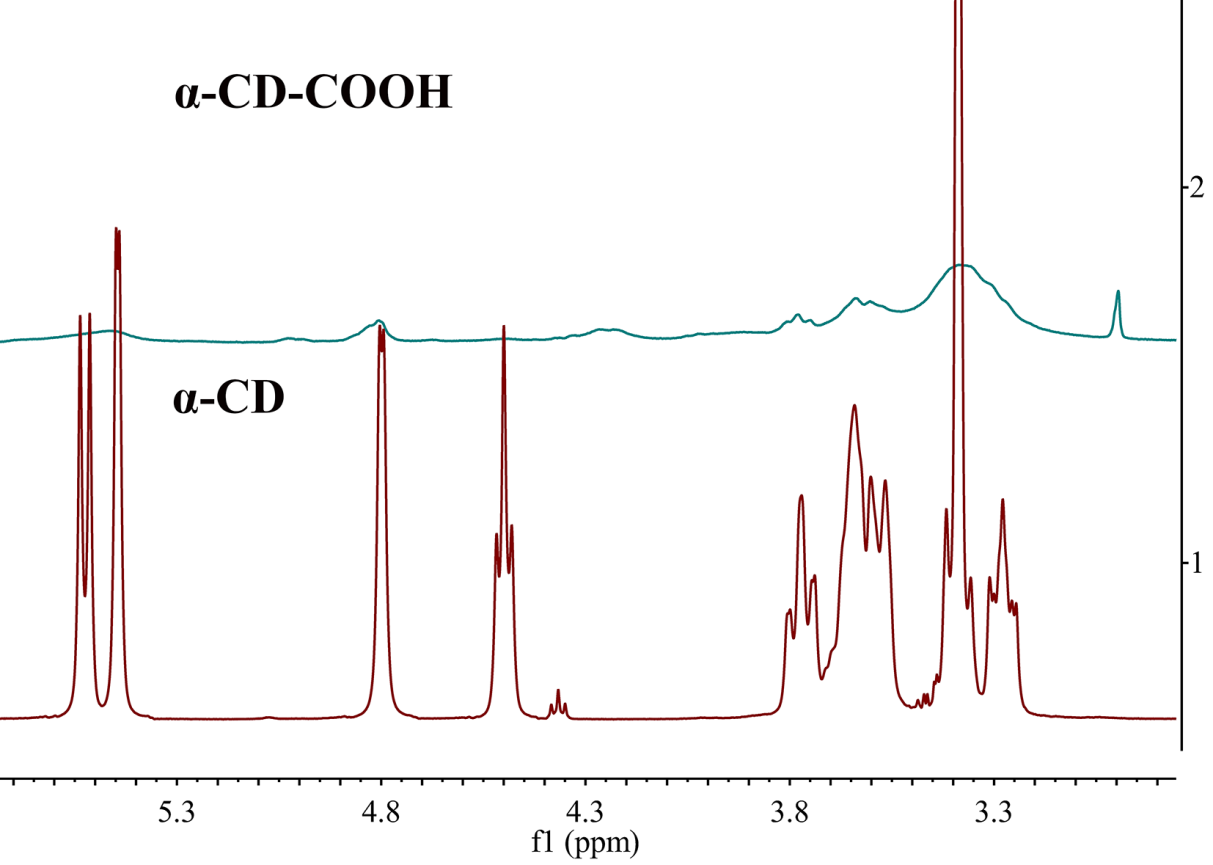


Fig. S1. The ^1^H NMR characterization of α-CD and α-CD-COOH.


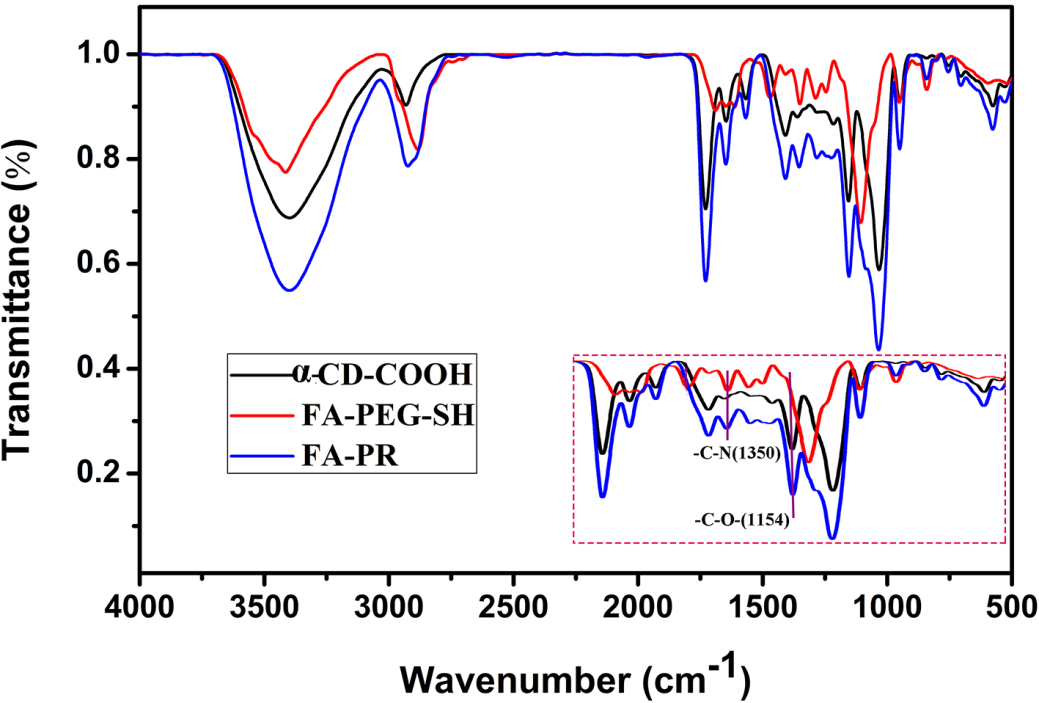


Fig. S2 FT-IR characterization of α-CD-COOH, FA-PEG-SH and FA-PR-SH.


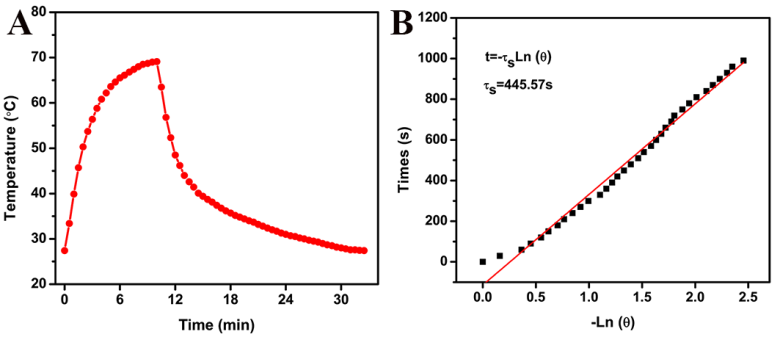


Fig. S3. The photothermal (A) and natural cooling curve (B) of AuNR@FA-PR/PEG/CDDP


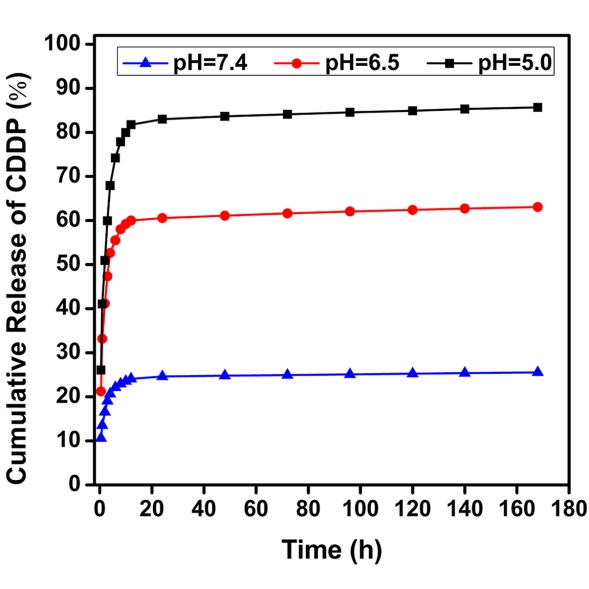


Fig. S4. The drug release profiles of AuNR@FA-PR/PEG/CDDP with an 808 nm laser (1.5 W cm^-2^) at different pH values.





Fig. S5. The cytotoxicity of CDDP and AuNR@FA-PR/PEG/CDDP to HL-7702 cells irradiated with and without an 808 nm laser (1 W cm^-2^).


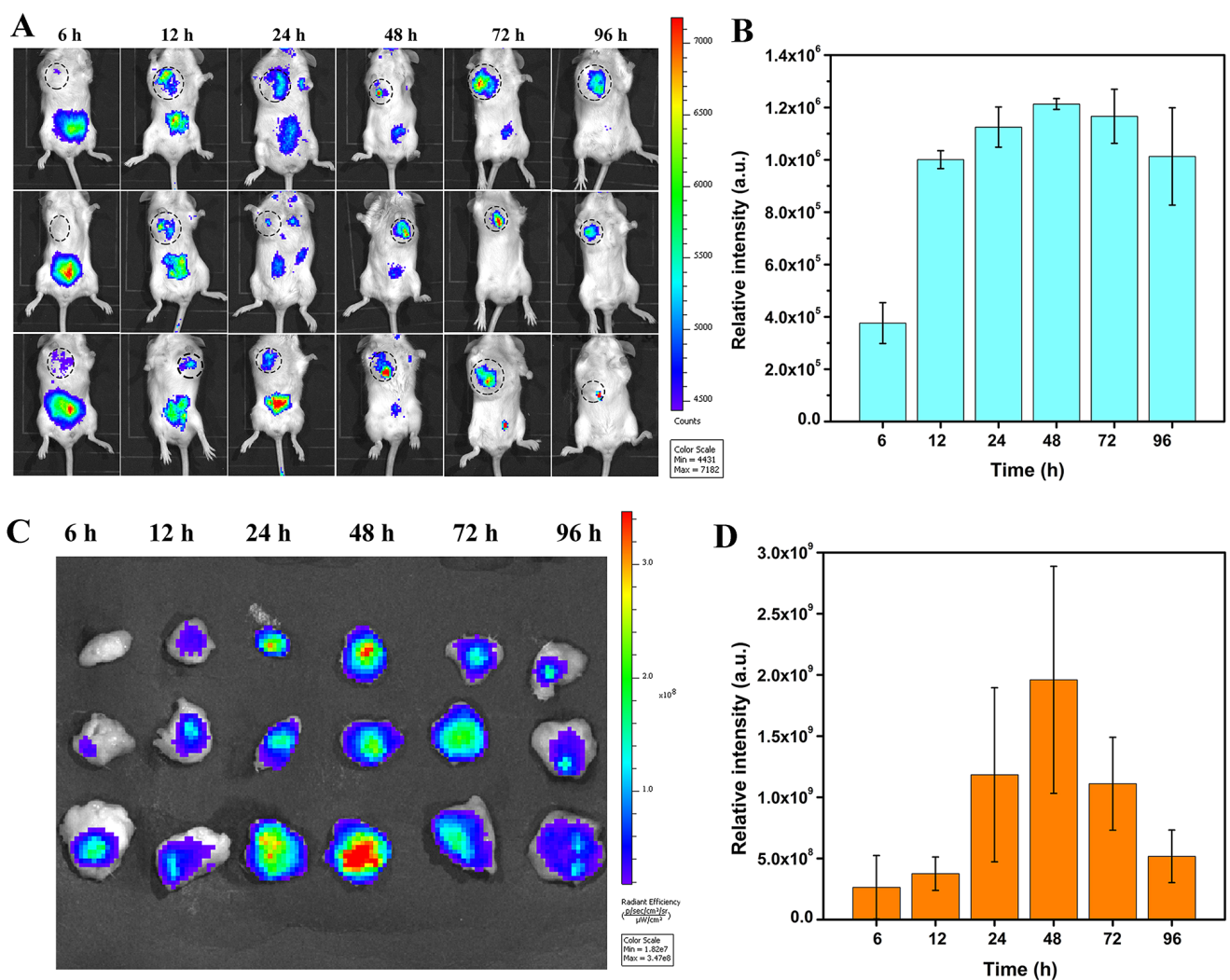


Fig. S6 (A) In vivo fluorescence imaging; (B) the fluorescence intensity of tumor area; (C) fluorescence time-imaging of tumors in dissected tumor at different time points; (D) the fluorescence intensity of dissected tumor at different time points





Fig. S7 AuNR@FA-PR/PEG/CDDP content in tumors at different time points. The unit is a percentage of injection doses per gram organ (% ID/g) versus time post-injection


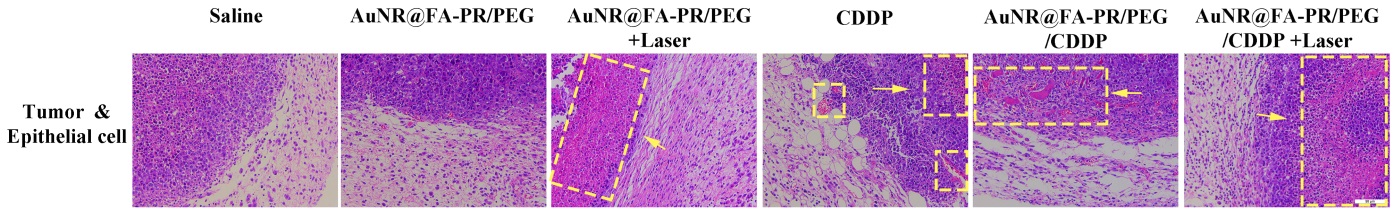


Fig. S8 H&E staining of the tumor and nearby tissues
